# Nanoparticle-Enabled Enrichment of Longitudinal Blood Proteomic Fingerprints in Alzheimer’s Disease

**DOI:** 10.1021/acsnano.1c00658

**Published:** 2021-03-17

**Authors:** Marilena Hadjidemetriou, Jack Rivers-Auty, Lana Papafilippou, James Eales, Katherine A. B. Kellett, Nigel M. Hooper, Catherine B. Lawrence, Kostas Kostarelos

**Affiliations:** † Nanomedicine Lab, School of Health Sciences, Faculty of Biology, Medicine and Health, The University of Manchester, Manchester M13 9PT, United Kingdom; ‡ Division of Neuroscience and Experimental Psychology, School of Biological Sciences, Faculty of Biology, Medicine and Health, The University of Manchester, Manchester Academic Health Science Centre, Manchester M13 9PT, United Kingdom; § Division of Cardiovascular Sciences, School of Medical Sciences, Faculty of Biology, Medicine and Health, The University of Manchester M13 9PT, Manchester, United Kingdom

**Keywords:** Alzheimer’s disease, early detection, biomarkers, protein corona, liposomes, nanomedicine, neurodegeneration

## Abstract

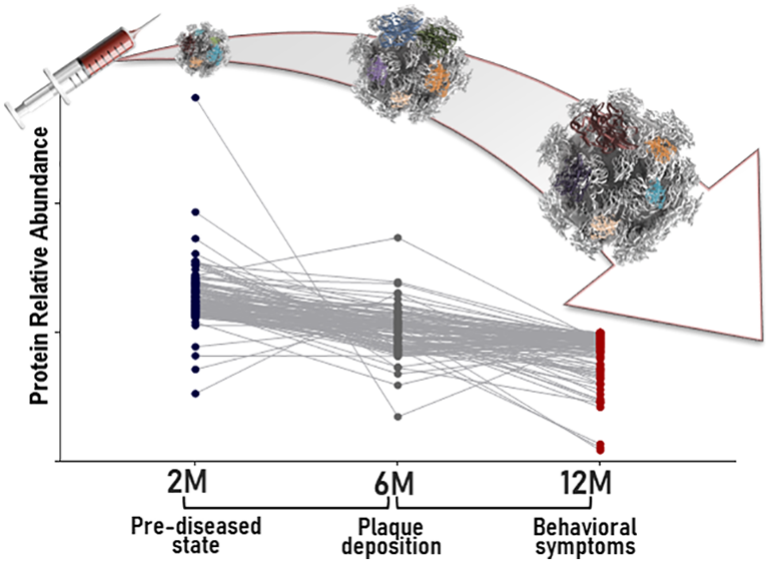

Blood-circulating biomarkers have the potential to detect Alzheimer’s disease (AD) pathology before clinical symptoms emerge and to improve the outcomes of clinical trials for disease-modifying therapies. Despite recent advances in understanding concomitant systemic abnormalities, there are currently no validated or clinically used blood-based biomarkers for AD. The extremely low concentration of neurodegeneration-associated proteins in blood necessitates the development of analytical platforms to address the “signal-to-noise” issue and to allow an in-depth analysis of the plasma proteome. Here, we aimed to discover and longitudinally track alterations of the blood proteome in a transgenic mouse model of AD, using a nanoparticle-based proteomics enrichment approach. We employed blood-circulating, lipid-based nanoparticles to extract, analyze and monitor AD-specific protein signatures and to systemically uncover molecular pathways associated with AD progression. Our data revealed the existence of multiple proteomic signals in blood, indicative of the asymptomatic stages of AD. Comprehensive analysis of the nanoparticle-recovered blood proteome by label-free liquid chromatography–tandem mass spectrometry resulted in the discovery of AD-monitoring signatures that could discriminate the asymptomatic phase from amyloidopathy and cognitive deterioration. While the majority of differentially abundant plasma proteins were found to be upregulated at the initial asymptomatic stages, the abundance of these molecules was significantly reduced as a result of amyloidosis, suggesting a disease-stage-dependent fluctuation of the AD-specific blood proteome. The potential use of the proposed nano-omics approach to uncover information in the blood that is directly associated with brain neurodegeneration was further exemplified by the recovery of focal adhesion cascade proteins. We herein propose the integration of nanotechnology with already existing proteomic analytical tools in order to enrich the identification of blood-circulating signals of neurodegeneration, reinvigorating the potential clinical utility of the blood proteome at predicting the onset and kinetics of the AD progression trajectory.

## Introduction

Alzheimer’s disease (AD) is a neurodegenerative disorder that results in a progressive and irreversible loss of memory and cognition.^1^ As life expectancy increases, the global economic and social burden of AD is expected to accelerate. However, currently there is no effective disease-modifying therapy for AD and existing pharmacological treatments are solely used to ameliorate symptoms.^2^

The implementation of effective treatments that can directly target the underlying mechanism of AD has largely failed so far, mainly due to the lack of early diagnostic tools.^2^ By the time symptoms emerge, the pathology is already well-established in the brain with the accumulation of amyloid-β plaques preceding cognitive symptoms by 10–15 years.^1^ Stratification biomarkers that can detect the asymptomatic onset of AD could dramatically improve the outcomes of clinical trials for disease-modifying therapies, which are expected to be more efficacious at the earlier stages of the AD continuum. Interest is also increasing rapidly in the development of surrogate biomarkers—indicators of AD progression that can be used as clinical end points—however, this currently remains a major challenge.^2,3^

Amyloid-β deposition in the brain is the most clinically established diagnostic and disease-monitoring marker and is currently assessed by positron-emission tomography (PET).^4^ In addition, measurements of amyloid-β and tau protein levels in the cerebrospinal fluid (CSF) are clinically used to aid AD diagnosis, alongside standard cognitive assessments. While CSF collection is relatively invasive, imaging modalities are expensive and therefore impractical for early diagnosis at the asymptomatic phase of AD. Hence, the focus is now turning toward the discovery of minimally invasive AD-specific signatures in blood, which could potentially track AD from the preclinical phase to the prodromal phase of mild cognitive impairment (MCI) and the onset of dementia.^5^

Although conventionally considered as a central nervous system (CNS) disorder, there is now increasing evidence that AD coexists with systemic abnormalities directly associated with underlying disease processes.^6^ In addition to the systemic manifestations observed, blood–brain barrier breakdown has been shown to be an early indicator of cognitive dysfunction reinvigorating the discovery of peripheral biomarkers for CNS disorders.^7^

Despite recent progress in the analysis of amyloid-β, tau, and neurofilament light chain in blood,^8−10^ there is no AD-specific blood biomarker that has gone beyond the discovery phase to validation. In addition to the obstacles associated with blood biomarker discovery, the extremely low concentration of neurodegeneration-associated proteins in blood, together with the large dynamic range of proteins and the masking effect of albumin, makes the discovery of AD-specific biomarkers extremely challenging.^11^ While a few studies have previously attempted to analyze the blood proteome of AD patients,^12^ the limited access to clinical samples at the asymptomatic stages has hampered the identification of early diagnostic biomarkers. Despite the conceptualization of AD as a biological and clinical continuum, most studies have so far attempted to discover molecular biomarkers at a single stage of the disease.^13,14^ Considering the limitations of currently available proteomic platforms, the discovery of blood biomarkers that can predict the temporal path of AD requires the development of proteomic analytical tools.

In this study, we aimed to identify and track longitudinal alterations of the blood proteome in a transgenic mouse model of AD (before and after the onset of plaque formation and cognitive impairment) using a nanotechnology-enabled approach. We have previously shown that the spontaneous surface-capture of hundreds of proteins by nanoparticles upon incubation with biological fluids (also known as “protein corona” formation) can be implemented as a tool for an in-depth analysis of the plasma proteome.^15−18^ Protein corona composition has been shown to reflect not only the differences in the plasma proteome observed between healthy and diseased states but also the differences observed between different healthy subjects.^19^

Here, we employed the nanoparticle protein corona as a tool to systematically monitor changes in the plasma proteome with AD progression and to reveal underpinning molecular mechanisms. It is now well-established that the binding affinity of nanoparticles with plasma proteins is determined by their physicochemical properties and therefore protein corona composition varies among different nanoparticles.^20−22^ The workflow of this study involved the intravenous administration and recovery of lipid-based nanoparticles from the blood circulation of APPswe/PS 1dE9 and wild-type (WT) control mice at 2, 6, and 12 months of age. Clinically used liposomes were employed because of their established pharmacokinetic and safety profile, their colloidal stability upon interaction with plasma components, and finally their efficient recovery and purification from the blood circulation of mice.^18,21,23^

Subsequent comparison of the resultant protein coronas by high-resolution liquid chromatrography−tandem mass spectrometry (LC-MS/MS) enabled the discovery of disease-specific signatures in blood, even at the earliest time point (before Aβ plaque formation). The relative abundance of the AD-specific proteins fluctuated over the course of the disease, indicating variations of the plasma proteome as the disease progresses. Our data demonstrate that while the majority of differentially abundant plasma proteins were found to be upregulated at the initial asymptomatic stages of the disease, the abundance of these molecules was significantly reduced as a result of amyloidosis and neurodegeneration, signifying the necessity for further longitudinal investigations of the blood proteome along the AD spectrum.

## Results and Discussion

### Blood-Circulating Nanoparticle Scavengers

**Figure 1:**
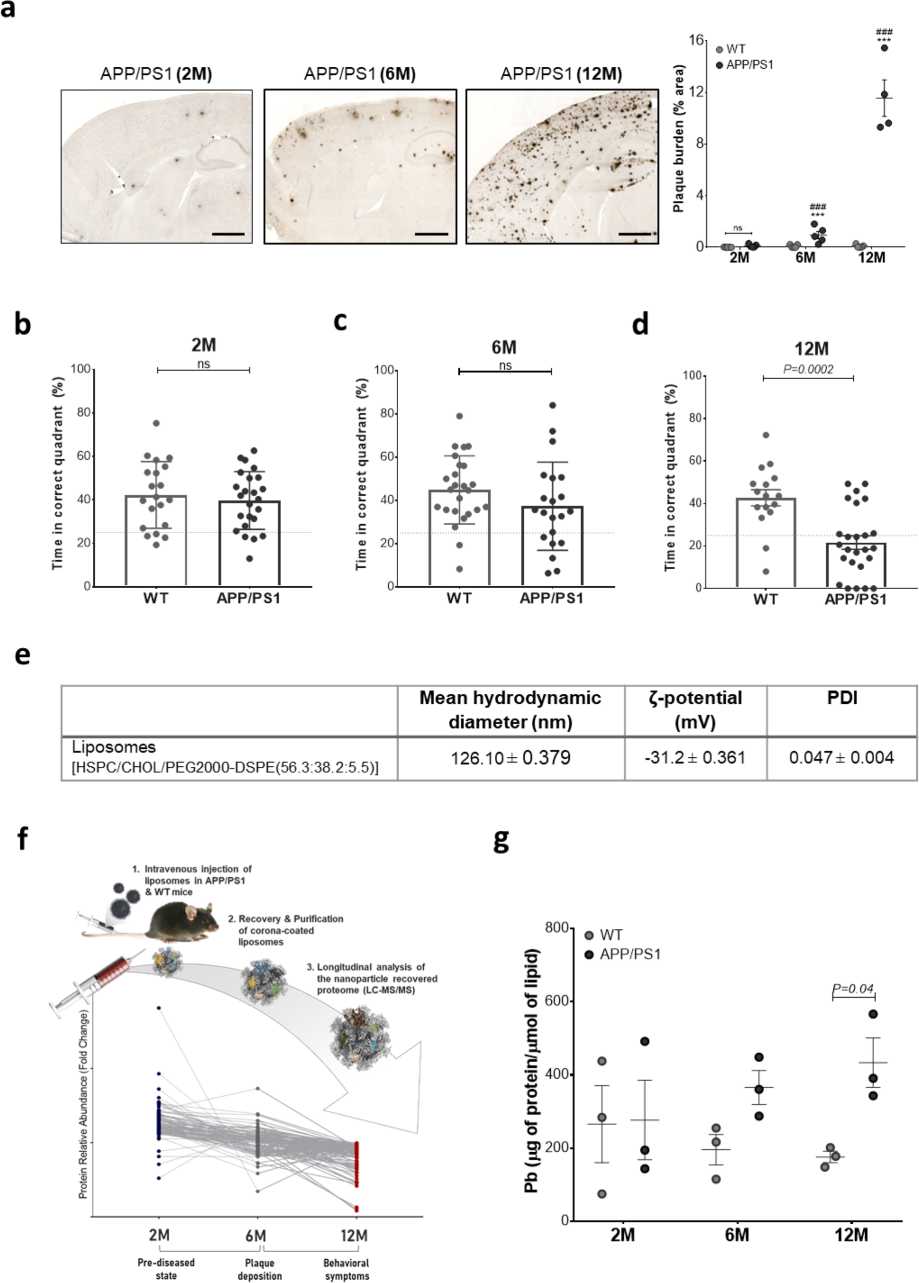
Blood-circulating nanoparticle scavengers in APP/PS1 and WT mice. (a) Quantification of Aβ plaque burden (percentage cortical area) in APP/PS1 and wild-type (WT) mice (C57BL/6j, male) at 2, 6, and 12 months of age with example images; cortical plaque deposition visualized with 6e10 antibody for Aβ 3–8. (Scale bars, 1 mm; Sidak corrected *post hoc* test; *** *p* value < 0.001, APP/PS1 *vs* WT at the same time point; ### *p* value < 0.001, APP/PS1 *vs* APP/PS1 at the previous time point; ns, not significant; *n* = 4–5). (b–d) Reference memory performance of APP/PS1 and WT (C57BL/6j) mice at 2, 6, and 12 months of age in the Morris water maze as measured by time (s) in the correct quadrant during the 30 s probe trial. Error bars indicate mean ± SEM (*n* = 19–25 mice, Welch’s test). 25% line represents expected performance from random chance. (e) Physicochemical characteristics of liposomes employed in this study. (f) Schematic description of the experimental design. PEGylated liposomes were intravenously injected and subsequently recovered from the blood circulation of 2, 6, and 12 month old APP/PS1 and wild-type C57 male mice. “Healthy” and “diseased” *in vivo* formed protein coronas were comprehensively characterized and compared by label-free mass spectrometry (LC-MS/MS) to identify differentially abundant proteins. (g) Total amount of protein adsorbed onto the surface of the blood-recovered liposomes, expressed as Pb values (μg of protein/μM lipid). Error bars indicate mean ± SEM of *n* = 3 biological replicates; *n* = 3 mice/replicate (Mann–Whitney *t* test).

The double transgenic mouse model of AD, APPswe/PS 1dE9,^24^ was employed, and plaque deposition and memory deficits were assessed in APP/PS1 and WT control mice at 2, 6, and 12 months of age utilizing the amyloid-β (6e10 antibody) staining and the Morris Water Maze (MWM) test, respectively (Figure 1a–d). In agreement with previous studies,^25^ nominal plaque deposition was observed at the 2 month time point, and no significant effects on memory were detected, which corresponds to a prediseased state (Figure 1a,b). The 6 month time point revealed statistically significant but mild plaque burden and no memory deficits, modeling the period between the pathophysiological manifestations of AD-related amyloidopathy and cognitive symptoms of the disease (Figure 1a,c), as described by the Jack *et al.* 2013 model of AD progression.^26^ As expected, the 12 month time point corresponded to symptomatic AD with significant plaque burden and substantial memory deficits (Figure 1a,d).

Considering the molecularly richer nature of the *in vivo*-formed protein corona as opposed to its counterpart *ex vivo* corona,^27^ PEGylated liposomes (HSPC:Chol:DSPE-PEG2000) were intravenously injected (*via* the tail vein) and subsequently recovered by cardiac puncture (10 min postinjection) from the blood circulation of 2, 6, and 12 month old APP/PS1 and WT C57BL/6 male mice (*n* = 3 mice/replicate; 3 independent biological replicates). For each biological replicate, plasma samples obtained from three mice were pooled together for a final volume of 1 mL. This not only ensures adequate concentration of recovered corona-coated liposomes but also minimizes any mouse-to-mouse variation of the plasma proteome.

The physicochemical characteristics of the liposomes employed are summarized in Figure 1e. Corona-coated liposomes were purified from any unbound plasma components by a two-step purification protocol which is based on size exclusion chromatography followed by membrane ultrafiltration, as previously extensively optimized and described.^15,16,21,23,27−29^ This protocol has been previously shown to completely eliminate unbound proteins and to result in a reproducible composition of protein corona.^18^ The resultant purified *in vivo* protein coronas at the three different time points were comprehensively characterized and compared (Figure 1f).

Negative stain transmission electron microscopy (TEM) revealed intact blood-recovered, protein-coated liposomes (Supporting Information Figure S1). To quantitatively compare the total amount of protein adsorbed onto the surface of liposomes at the three different time points of investigation, bicinchoninic acid (BCA) protein assay was performed and protein-binding (Pb) values were calculated (expressed as μg of protein/μmol of lipid). As shown in Figure 1g, the average Pb value increased with age only in the APP/PS1 mice, while significant changes were observed between APP/PS and WT mice only at the 12 month time point. These results suggest that protein corona fingerprints quantitatively differ as a result of amyloidopathy and cognitive impairment.

### AD-Specific Longitudinal Proteomic Alterations in Blood

The goal of the proteomic discovery experiment was to longitudinally monitor and compare the blood proteome of APP/PS1 and WT mice, in order to capture molecular changes indicative of AD pathophysiology. Equal amounts of total protein from plasma samples (without prior incubation with liposomes) and corona samples (upon *in vivo* recovery and purification of intravenously injected liposomes) were trypsin-digested and subsequently analyzed by LC-MS/MS. It should be noted that highly abundant proteins (*e.g.*, albumin and immunoglobulins) were not depleted from plasma and corona samples prior to LC-MS/MS analysis. The extensive purification of unbound plasma proteins from corona samples has been shown to reproducibly increase the range of proteins detected, enabling the identification of low molecular weight and low abundance proteins.^15,16^ Thus, in the case of the corona samples only proteins with high affinity for the liposomal surface or smaller proteins carried by other proteins directly adsorbed onto the liposome surface were analyzed.

**Figure 2:**
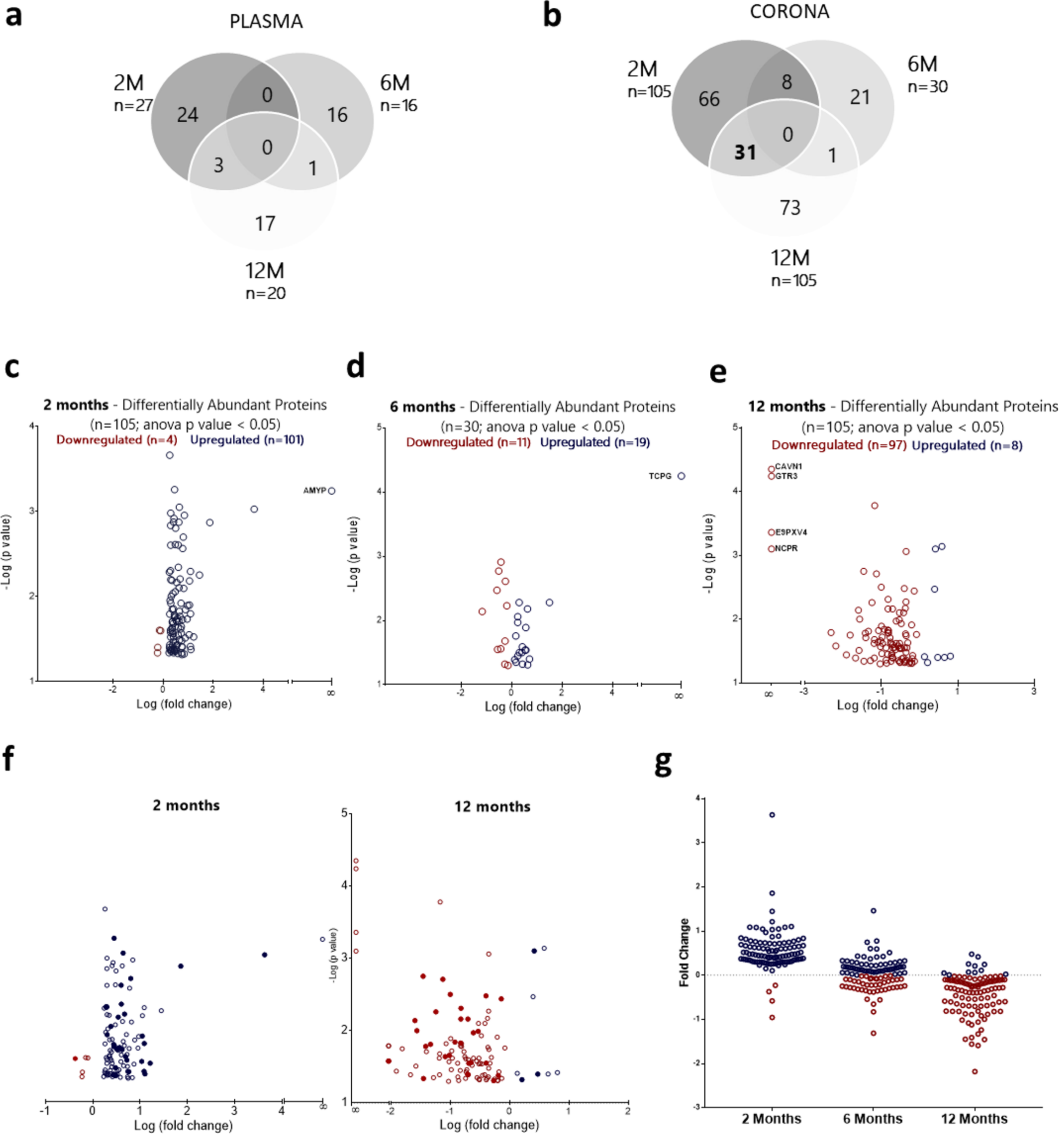
AD-specific longitudinal proteomic alterations in blood. Proteomic comparison between “healthy” and “diseased” protein coronas at the 2 month (2M), 6 month (6M), and 12 month (12M) time points (*n* = 3 biological replicates; *n* = 3 mice/replicate). MS peak intensities were analyzed using Progenesis LC-MS software (v. 3.0; Nonlinear Dynamics). Only proteins that differed between APP/PS1 and WT mice with a *p* value < 0.05 are shown. Venn diagrams report the number of differentially abundant proteins discovered at the three time points (2M, 6M, and 12M) by proteomic analysis of (a) plasma control samples and (b) corona samples. (c–e) Volcano plots display the relationship between fold change and significance for the differentially abundant corona proteins at 2M, 6M, and 12M. The full list of differentially abundant proteins is shown in Supporting Information Tables S1–S3. (f) Comparison of the differentially abundant proteins discovered at 2M and 12M. Filled dots represent the *n* = 31 common proteins between the two time points. (g) Longitudinal fluctuation in the fold change values of the *n* = 105 proteins identified to be differentially abundant between APP/PS1 and WT mice at the 2M time point.

Processing of the proteomic data generated with Progenesis QI (v. 3.0; Nonlinear Dynamics) software was carried out in order to statistically compare the relative protein expression (fold change) and reliability of measured differences between the blood proteome in APP/PS1 and WT mice. The Venn diagrams of Figure 2a,b illustrate the number of differentially abundant proteins at the three different time points of investigation, as identified by proteomic analysis of plasma and corona samples, respectively. A significantly higher number of differentially abundant proteins were detected in the corona samples in comparison to the number of proteins identified by plasma control analysis for all three time points of investigation (Figure 2a,b). This agrees with our previously published work and elucidates the need for analytical platforms that can uncover disease-associated molecules in blood, otherwise masked by the predominant signal of albumin.^15,16^

As shown in Figure 2b, multiple differentially abundant proteins (*n* = 105) were identified between APP/PS1 and WT mice even at the earliest time point of investigation, suggesting that alterations of the blood circulatory proteome may occur at the asymptomatic phase of AD. None of the differentially abundant proteins discovered were found to be common for all three time points, which demonstrates that the composition of the protein corona is directly affected by the disease stage. A total number of 66 differentially abundant proteins were exclusively found at the earliest stage of AD (2 months), while *n* = 21 and *n* = 73 proteins were exclusively found at the intermediate (6 months) and late (12 months) phases of AD development, respectively. The full lists of differentially abundant proteins are shown in Supporting Information Tables S1–S3. Interestingly, clusterin (apolipoprotein J), one of the most promising candidate blood biomarkers identified in multiple independent discovery studies as an early indicator of amyloid deposition,^30^ was found in our study to be upregulated only at the 2 month time point.

Among the differentially abundant proteins identified by comparing the protein coronas formed in APP/PS1 and WT mice, pancreatic alpha amylase (Amy2) and T-complex protein 1 (Cct3) were exclusively identified in APP/PS1 mice at 2 and 6 months of age, respectively, while caveolae-associated protein 1 (Cavin1), solute carrier family 2 facilitated glucose transporter member 3 (Slc2a3), bile acyl-CoA synthetase (Slc27a5), and NADPH-cytochrome P450 reductase (Por) were only identified in WT mice and were completely absent in the corona samples recovered from 12 -month old APP-PS1 mice (Figure 2c–e). The “presence” or “absence” of certain proteins from the coronas formed in AD mice in comparison to control mice of the same age indicate clear differences between the two groups which further supports our hypothesis that blood-circulating liposomes can capture molecular changes indicative of AD pathophysiology.

Given the co-occurrence of numerous systemic abnormalities in AD, monitoring of multiple blood analytes is needed to collectively reflect AD-related processes in the brain. Distribution of the corona proteins identified by statistical significance and magnitude of change revealed that while the majority of differentially abundant proteins were upregulated in APP/PS1 mice in comparison to WT mice at the 2 month time point, 97 out of 105 differentially abundant proteins were found to be downregulated at the 12 month time point (Figure 2c–e).

The above observation prompted us to further investigate the kinetics of the blood alterations as the disease progresses. A closer comparison between the 2 and 12 month time points revealed 31 common proteins with differential abundance between APP/PS1 and WT mice, of which 26 were upregulated at the earliest time point and gradually became downregulated, as pathophysiological changes culminated in cognitive impairment (Figure 2f and Supporting Information Figure S2). Interestingly, this downregulation effect was observed for the majority of the upregulated proteins identified at the 2 month time point. As illustrated in Figure 2g, the progressive increase of Aβ plaque deposits in the cortex and hippocampus of APP/PS1 mice resulted in the gradual reduction in the blood concentration of the proteins upregulated at 2 months. This explains the significantly lower number (*n* = 30) of differentially abundant proteins identified at the intermediate stages of AD progression and indicates that the development of brain amyloidopathy is systemically mirrored beyond the brain.

### Systemic Monitoring of AD Progression

The above proteomic comparison between the liposomal coronas formed in APP/PS1 and WT mice suggested that intravenously administered nanoparticles can capture numerous systemic signatures that reflect AD-related processes in the brain even at the onset of AD. To assess the enrichment of AD-monitoring proteomic signatures in blood, which could distinguish the asymptomatic phase from mild amyloidopathy and cognitive deterioration, we further investigated the temporal evolution of corona formation in APP/PS1 mice at 2, 6, and 12 months of age, by statistically comparing the respective corona profiles.

**Figure 3:**
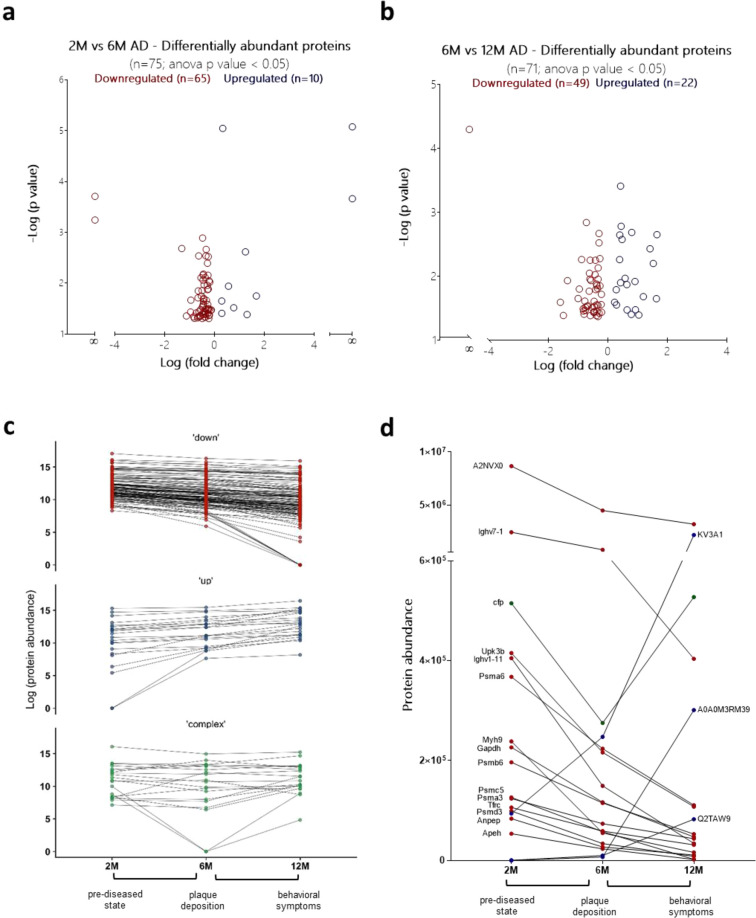
Systemic monitoring of AD progression. Time evolution of the liposomal coronas in APP/PS1 mice. Proteomic comparisons between (a) 2M *vs* 6M and (b) 6M *vs* 12M time points (*n* = 3 biological replicates; *n* = 3 mice/replicate). MS peak intensities were analyzed using Progenesis LC-MS software (v. 3.0; Nonlinear Dynamics). Only proteins that differed by a *p* value < 0.05 are shown. Proteins that were differentially abundant between 2M *vs* 6M and 6M *vs* 12M WT mice as a result of aging were excluded and only AD-specific-monitoring proteins are shown. The volcano plot displays the relationship between fold change and significance between the two groups. The full lists of potential biomarker proteins are shown in Supporting Information Tables S4 and S5. Longitudinal kinetics in the abundance of (c) all the disease-monitoring proteins identified and (d) the *n* = 18 common proteins that displayed differential abundance between 2M and 6M and between 6M and 12M time points. Proteins are classified in three groups: proteins with increased abundance with AD progression (shown in blue), proteins with decreased abundance with AD progression (shown in red), and proteins characterized by more complex kinetics (shown in green).

As illustrated in the volcano plots of Figure 3a,b, proteomic comparison of the “diseased” coronas at the three different time points revealed statistically significant differences. While 75 proteins were differentially abundant between the 2 and 6 month time points (Figure 3a), 71 proteins were found to be differentially abundant between the 6 and 12 month time points (Figure 3b). It should be noted that “healthy” corona profiles were also compared between WT mice of different age as a control, in order to exclude any aging-related differences and to identify only AD-specific-monitoring proteins.

To gain some further understanding of the protein-binding kinetics as AD progresses, we classified the above differentially abundant proteins into three groups according to the fluctuation of their normalized protein abundance value over time: (a) proteins with increased abundance with AD progression, (b) proteins with decreased abundance with AD progression, and (c) proteins characterized by lower abundance at the prediseased state (2 months) and at later time points (12 months), but displaying peak abundance at the intermediate state (6 months) or *vice versa*. As depicted in Figure 3c, the majority of the disease-altered proteins identified displayed decreased plasma levels with disease progression, which in some cases resulted in the complete absence of these proteins from the corona samples recovered from 12 month old APP/PS1 mice. It should be noted that the majority of downregulated and upregulated proteins displayed a linearly altered abundance with disease progression (Figure 3c).

Among the above AD-stage-specific protein signals, we identified a group of 18 proteins which could differentiate not only the prediseased state from mild plaque burden but could also discriminate cognitive deterioration (Figure 3d). Fourteen out of the 18 proteins displayed a gradual reduction in their abundance with disease progression, while only 3 exhibited increased abundance. Interestingly, inverse peak-shaped abundance kinetics were observed for properdin (cfp), a component of the alternative complement pathway previously associated with plaque deposition in transgenic mouse models of AD (Figure 3d).^31^

Collectively, our findings here reveal AD-stage-specific alterations of the plasma proteome. The complex kinetics of the plasma proteome observed (Figure 3c) suggest a direct connection between the brain neurodegeneration and the blood proteome, which necessitates the need for longitudinal rather than cross-sectional biomarker discovery studies.

### Molecular Pathway Enrichment Analysis

**Figure 4:**
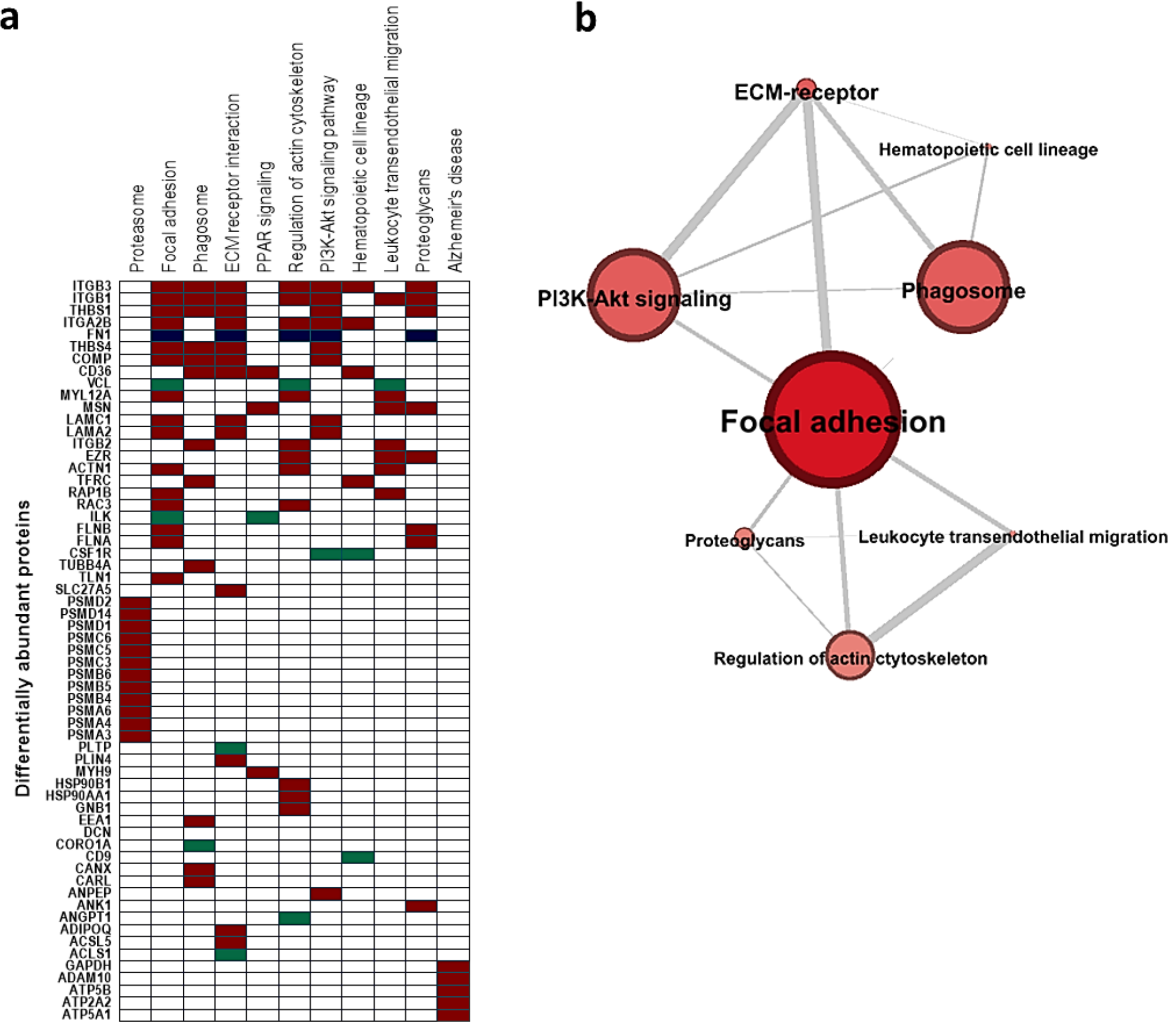
Molecular pathway enrichment analysis. (a) Clustegram illustrating the 10 most enriched pathways (columns) from Kyoto Encyclopedia of Genes and Genomes (KEGG) and the 56 differentially abundant blood proteins involved (rows). Proteins are ranked from high to low frequency, and pathways are ranked according to the significance level of enrichment. The proteins identified to be involved in the KEGG Alzheimer’s disease pathway are also shown. Proteins with increased abundance with AD progression are shown in blue, proteins with decreased abundance with AD progression are shown in red, and proteins characterized by more complex kinetics are shown in green. (b) Protein interaction network. Nodes, representing the 10 most enriched pathways, are sized according to the number of constituent proteins. Connections between nodes are sized according to the number of shared proteins between the pathways. .

In order to gain some insight into the molecular pathways that were activated systemically in response to amyloidopathy, we performed pathway enrichment analysis for all the differentially abundant proteins identified between APP/PS1 and WT mice, using the Enrichr’s analysis tool. Proteins were classified by Kyoto Encyclopaedia of Genes and Genomes (KEGG) database. As shown in Supporting Information Table S6, the identified differentially abundant proteins were found to act in 32 major pathways (adjusted *p* value < 0.05). Proteasome (*p* value = 5.29 × 10^–15^), focal adhesion (*p* value = 3.50 × 10^–13^), phagosome (*p* value = 1.27 × 10^–10^), ECM–receptor interaction (*p* value = 3.89 × 10^–9^), PPAR signaling (*p* value = 3.80 × 10^–7^), regulation of actin cytoskeleton (*p* value = 6.78 × 10^–7^), PI3K-Akt signaling (*p* value = 9.73 × 10^–7^), hematopoietic cell lineage (*p* value = 3.04 × 10^–06^), leukocyte transendothelial migration (*p* value = 8.91 × 10^–6^), and proteoglycans (*p* value = 1.68 × 10^–5^) were found to be the 10 most significantly enriched pathways (Figure 4a,b).

Moreover, the following five proteins were found to be directly involved in the AD KEGG pathway (Figure 4a), namely, glyceraldehyde-3-phosphate dehydrogenase (GAPDH), disintegrin, and metalloproteinase domain-containing protein 10 (ADAM10), ATP synthase subunit beta, mitochondrial (ATP5B), sarcoplasmic/endoplasmic reticulum calcium ATPase 2 (ATP2A2), and ATP synthase subunit alpha, mitochondrial (ATP5A1). All five proteins were identified to be downregulated in APP/PS1 mice in comparison to WT mice at the 12 month time point, and three of them (ADAM10, ATP5B, and ATP2A2) were also found to be upregulated at the 2 month time point (Supporting Information Tables S1 and S2).

As illustrated in Figure 4a, the majority of proteins associated with the 10 most enriched pathways displayed a decreased abundance with disease progression. Among the differentially abundant proteins, integrin beta-3 (Itgb3) and integrin beta-1 (Itgb1) were found to be the most frequently identified proteins, involved in 7 out of the 10 most enriched pathways. Interestingly, cellular component enrichment analysis revealed that nine proteins were constituents of myelin sheath (ATP synthase subunit beta, mitochondrial, ATP5B; ATP synthase subunit alpha, mitochondrial, ATP5A1; calnexin, CANX; guanine nucleotide-binding protein G(I)/G(S)/G(T) subunit beta-1, GNB1; tubulin beta-4A chain, TUBB4A; ezrin, EZR; moesin, MSN; integrin beta-1, Itgb1; and heat shock protein HSP 90-alpha, HSP90AA1).

On the basis of the above analysis, a protein interaction network was constructed using the Gephi visualization platform. As illustrated in Figure 4b, the focal adhesion pathway, previously identified to play a key role in synaptic plasticity and activity,^32^ was identified as the central node with 18 differentially abundant proteins being involved.

In this work, we identified and longitudinally tracked alterations of the blood proteome in a transgenic mouse model of AD, using intravenously administered nanoparticles as an “omics” enrichment analytical platform.

The blood circulatory proteome is likely to echo the complex cascade of molecular pathways associated with AD progression. Given the disruption of the BBB in AD and the increased permeability of molecules as a result, ongoing efforts are focused on the discovery of blood proteomic signatures that can non-invasively describe the molecular pathogenesis of AD.^7^ Over the past decade significant progress has been made in the systemic detection of AD, with multiple high throughput omics studies reporting a range of blood-based biomarkers that differ between AD and control subjects,^33,34^ yet there are not any validated or clinically used blood-based biomarkers for AD.^35^

Among the blood-circulating analytes, to date, proteins have been mostly studied for AD diagnosis. However, their identification by mass spectrometry is hindered by the molecular complexity of the blood proteome, in addition to the “signal-to-noise” issue caused by albumin and immunoglobulins. Brain-derived proteins are subjected to massive dilution and rapid degradation in the blood circulation which makes their detection even more challenging.^12^ In addition to the above technical issues, systemic inflammation, multimorbidity, and polypharmacy are common features in AD patients that further compromise the identification of highly specific blood molecules.

Although a number of studies have investigated the use of brain imaging to longitudinally monitor AD, the use of disease-monitoring molecular biomarkers has been so far limited to CSF measurements of amyloid-β and tau proteins.^36^ Moreover, only a few human clinical studies have previously investigated the longitudinal alterations of the blood proteome in AD, and they were mostly focused on the targeted quantification of already known biomarker candidates, rather than the discovery of previously unseen biomarkers.^8,9,12,37−39^ Of the known candidate biomarkers in plasma, total-tau, neurofilament light chain, and amyloid-β levels have been the most extensively studied as longitudinal measurements of AD monitoring.^40^

The aforementioned issues associated with the nontargeted discovery of neurodegeneration-associated proteins in blood mandate the development of proteomic analytical platforms. We have previously shown that the spontaneous surface capture of hundreds of proteins by nanoparticles upon their *in vivo* or *ex vivo* interaction with biological fluids (also known as “protein corona” formation) can be implemented as a tool for an in-depth analysis of the cancer plasma proteome.^15,16^ In the present study, we investigated the use of intravenously administered nanoparticles to extract and analyze blood protein patterns indicative of AD. We hypothesized that proteomic analysis of the nanoparticle-enriched proteome recovered from APP/PS1 and WT control mice would reveal differences in a time-dependent manner.

We investigated three different time points (2, 6, and 12 months) that model the prediseased state, the intermediate state between AD-related amyloidopathy and cognitive symptoms, and finally symptomatic AD (Figure 1a,b). Considering that human longitudinal studies are challenging due to the limited access to clinical samples at prediseased states,^25^ we chose to perform this proof-of-concept study using a transgenic mouse model of AD (APP/PS1), in order to demonstrate the feasibility of systemically monitoring AD and to provide some insight into the dynamic evolution of the plasma proteome with AD progression.

To prove our hypothesis, the nanoparticle-recovered blood proteome was subjected to LC-MS/MS analysis. The subsequent comparison of the resultant protein coronas, formed in APP/PS1 and WT control mice, revealed the identification of multiple disease-specific signatures in blood, even at the earliest time point (before Aβ plaque deposition). The advantage of using this nanoparticle enrichment approach was demonstrated by the significantly higher number of differentially abundant proteins identified by the analysis of the corona samples in comparison to plasma control analysis (Figure 2a,b). The distinct proteomic fingerprints observed at the three different time points of investigation (before and after plaque formation and cognitive impairment; Figure 2c–e) suggest a clear connection between the nanoparticle-harvested proteome and the disease development in the brain.

Interestingly, although the majority of differentially abundant proteins were found to be upregulated in APP/PS1 mice in comparison to WT mice at the asymptomatic stage, 97 out of the 105 differentially abundant proteins identified at the late symptomatic stage were found to be downregulated (Figure 2f). Prompted by the above observation, we monitored the temporal evolution of the differentially abundant proteins identified at the earliest time point and our data revealed an overall downregulation effect with disease progression (Figure 2g). The disease-stage-dependent fluctuation of the AD-specific blood proteome observed could explain the lack of reproducibly identified blood-based biomarkers for AD.^12^

Furthermore, to identify blood protein signatures that could systemically monitor amyloid burden and cognitive impairment, we statistically compared the blood proteome fingerprints recovered from APP/PS1 mice of 2, 6, and 12 months of age. We identified three kinetically defined groups of disease-monitoring proteins: (a) proteins with increased abundance with AD progression; (b) proteins with decreased abundance with AD progression; and (c) proteins characterized by lower abundance at the prediseased state (2 months) and at later time points (12 months) but displaying peak abundance at the intermediate state (6 months) or *vice versa* (Figure 3c). Interestingly, the majority of disease-monitoring proteins identified displayed decreased plasma levels with AD progression (Figure 3c,d). These findings could be explained by the increased permeability of the BBB and subsequent translocation of blood proteins into the brain tissue and/or by the compromised diffusion of molecules from the brain to the blood circulation, as a result of protein misfolding and aggregation.

The complex protein kinetics observed suggest a direct connection between the blood proteome and brain neurodegeneration, which necessitates the need for longitudinal rather than cross-sectional biomarker discovery studies.^41^ Considering the co-morbidities associated with neurodegeneration, it is now increasingly accepted that multiple biomarkers will be needed to provide adequate specificity and sensitivity for AD diagnosis. Our data demonstrate that the analysis of the nanoparticle protein corona has the potential to uncover and monitor the kinetics of multiple molecules in blood that reflect AD-related processes in the brain. Whether the above protein fingerprints identified in the plasma proteome of APP/PS1 mice could be clinically exploited as disease-monitoring biomarkers for AD is beyond the scope of this study and requires validation experiments using human clinical samples. Future validation studies should also consider the human-to-human variation in the plasma proteome which is reflected in the formation of a “personalized corona”, even in healthy individuals.^19^

The connection between the brain tissue and blood during AD progression and the subsequent bidirectional transport of cells and proteins across the BBB are still poorly understood.^41^ In addition to the accumulation of amyloid-β plaques and degeneration of memory, recent high throughput genomic approaches revealed a complex network of dysregulated pathways in the brain, including mitochondrial dysfunction, deficits in glucose availability, neuronal damage, synapse loss, and inflammatory activation of microglia and astrocytes.^32,42^ In order to gain some insight into the underpinning mechanisms of brain amyloidopathy that are reflected in the blood proteome, we performed molecular pathway enrichment analysis. Our results revealed the focal adhesion cascade as a central hub in the disease development (Figure 4a,b). This finding is in line with the recently proposed genetic landscape of AD, in which dysfunction of the focal adhesion pathway and the related cell signaling are key elements in AD pathogenesis.^32^

The potential use of intravenously injected nanoparticles to uncover information in the blood that is directly connected with the molecular cascade of neurodegeneration in the brain was further exemplified by the identification of five proteins that are involved in the AD pathway, namely, glyceraldehyde-3-phosphate dehydrogenase (GAPDH), disintegrin, and metalloproteinase domain-containing protein 10 (ADAM10), ATP synthase subunit beta, mitochondrial (ATP5B), sarcoplasmic/endoplasmic reticulum calcium ATPase 2 (ATP2A2), and ATP synthase subunit alpha, mitochondrial (ATP5A1) (Figure 4a). It should be noted that a significant decrease of ADAM10 in AD cerebrospinal fluid and platelets and an upregulation of GAPDH in circulating leukocytes have been previously reported; however, their use as potential plasma biomarkers has not been proposed and requires further investigation.^43−45^ Despite the documented significant role of the above-mentioned proteins in AD development, their simultaneous recovery from plasma, dynamic monitoring, and their potential value in systemically monitoring AD progression has not been previously shown.

## Conclusion

In this study, we propose the use of blood-circulating nanoparticles to extract, analyze and track blood proteomic signatures directly associated with amyloidopathy and neurodegeneration in the brain. We employed a transgenic mouse model of AD in order to identify and systemically monitor disease-specific protein fingerprints from the asymptomatic phase, to plaque deposition and cognitive deterioration. Our data suggest a strong correlation between the nanoparticle-extracted blood proteome and AD pathology, even at the prediseased state. The proposed nano-omics enrichment approach enabled the discovery of multiple AD-specific diagnostic and disease-monitoring blood proteomic patterns and revealed underlying molecular pathways involved in the AD pathophysiology, including the focal adhesion cascade. Monitoring of AD-associated proteins in blood revealed substantial fluctuation in their abundance with disease progression. Future longitudinal studies are needed to investigate the clinical utility of the blood proteome in predicting the onset and kinetics of a wide range of neurodegenerative disorders.

## Experimental Section

### Animals

APPswe PSEN1ΔE9 (APP/PS1) transgenic mice^24^ on a C57BL/6j background were obtained from the Jackson Laboratory (no. 005864) and bred to produce hemizygous APP/PS1 male mice and C57BL/6j (wild-type) littermates. All mice were housed under standard conditions at 22 ± 20 °C and a standard 12 h light/dark cycle with free access to food and water. All animal experiments were carried out in accordance with the United Kingdom Animals (Scientific Procedures) Act 1986 and approved by ethical committees under license no. P1D200E0B. Animals were coded in order to blind the individual evaluating the behavioral tasks and immunohistological sections.

### Immunohistochemistry

Mouse brains were split into hemispheres and the right hemisphere was immerse-fixed in 4% paraformaldehyde for 24 h. The hemispheres were then dehydrated and paraffin-embedded, and 5 μm sections were taken sagittally every 1 mm from the central sulcus and mounted onto Superfrost Plus slides (VWR). Antigen retrieval was performed by 30 min incubation in 0.2 mM citrate buffer pH 6 at 96 °C followed by 10 min immersion in 90% formic acid. Sections were then washed (3×, 5 min) with 0.1% tween in phosphate buffered saline (PBST) and blocked for 1 h in a 1% bovine serum albumin (BSA, A9647 Sigma-Aldrich), 0.2 M phosphate buffer (PB) solution. The slides were then incubated overnight at 4 °C in 1:200 biotinylated 6e10 antibody (no. SIG-39340-200, Covance) in a 1% BSA, 0.2 M PB solution. The sections were washed (3×, 5 min, PBST), exposed to 1:20 strep-avidin (P188503, RnD) 1% BSA, 0.2 M PB solution for 2 h at room temperature, washed, and then visualized with a DAB-nickel solution (D0426-50SET, Sigma-Aldrich). The slides were then dehydrated in ethanol and xylene, and then cover-slipped using DPX (DI5319/05, Fisher Scientific). The sections were then scanned, and the percentage stained area was calculated through threshold–particle analyses performed on ImageJ. The three sagittal sections 1 mm apart per mouse were processed and analyzed in this way.

### Morris Water Maze

Morris water maze (MWM) was used to evaluate memory deficits at the 2, 6, and 12 month time points in the APP/PS1 and WT mice. The tank was 1 m in diameter with a 10 cm platform and large visual cues in all directions. Water temperature was maintained at 22 ± 2 °C, and white noise (40 db) was on during habituation to the room and the MWM task. The MWM was performed as previously described^46^ with some modifications. A 6 day protocol was used consisting of habituation, a cued trial day, 4 trial days, and a probe trial day. Mice were placed in the behavior room during the entire period of the study.

Tracking analysis was performed on ANY-maze software. To ensure equal motivation and ability for the task, a cued trial was performed. A platform with a large black and white flag was placed in a random quadrant (NE, SE, NW, SW). Mice were placed in the water maze at a random starting location. The trial was stopped when the mouse found the platform or 60 s had lapsed, in which case the mice were guided to the platform. The mice were then dried and placed in warmed cages. This was repeated four times with new platform and starting locations and a minimum of 60 min between trials. During the trial days the hidden platform was placed in the NE quadrant of the maze. As above, mice were placed in the maze at a random starting location, given 60 s to find the platform, dried, and placed in warmed cages and four repeats were performed per day. The mice were tasked with learning the location of the hidden platform over four trial days. During the probe trial the hidden platform was removed. The mice were placed in the maze facing the wall and allowed to swim for 30 s. Swim speed, total distance traveled, time in each quadrant, number of entries in the platform zone, and a number of other factors were measured. Time (%) in the correct quadrant (NE) was our primary measure of memory performance.

### Preparation of Liposomes

HSPC:Chol:DSPE-PEG2000 (56.3:38.2:5.5) liposomes were prepared, by thin lipid film hydration method followed by extrusion, as previously described.^10,11^ The physicochemical properties of the liposomes employed were measured using Zetasizer Nano ZS (Malvern Instruments, U.K.) and are shown in Figure S1.

### *In Vivo* Administration of Liposomes

Liposomes were intravenously injected *via* the lateral tail vein (at a lipid dose of 0.125 mM/(g of body weight)) and subsequently recovered by cardiac puncture (10 min postinjection) from the blood circulation of 2, 6, and 12 month old APP/PS1 and WT mice. For each time point three biological replicates were performed. For each of these biological replicates, three mice were used (*n* = 3 biological replicates, *n* = 3 mice/replicate). The plasma samples obtained from three mice were pooled together for each biological replicate. Blood, containing corona-coated liposomes, was collected in K2EDTA-coated tubes. ∼0.5 mL of blood sample was collected from each mouse. Plasma was then prepared by centrifugation for 12 min at 1200 RCF at 4 °C after inverting the collection tubes to ensure mixing of blood with EDTA. Plasma was collected into Protein LoBind Eppendorf tubes. The plasma samples obtained from three mice were pooled together for a final plasma volume of 1 mL.

Corona-coated liposomes were separated from unbound and weekly bound plasma proteins by size exclusion chromatography followed by membrane ultrafiltration as previously described.^23,27^

### Transmission Electron Microscopy

Bare and corona-coated liposomes were stained with uranyl acetate (UA) solution 1% and visualized with transmission electron microscopy (FEI Tecnai 12 BioTwin) before and after their *in vivo* interaction with plasma proteins. Samples were diluted to 0.5 mM lipid concentration, and carbon film mesh copper grids (CF400-Cu, Electron Microscopy Science) were used.

### Quantification of Adsorbed Proteins

Proteins associated with recovered liposomes were quantified by BCA Protein assay kit according to the manufacturer’s instructions. To make sure that liposomes in solution do not interfere with the absorbance at 562 nm, the absorbance of corona-coated liposomes in HEPES buffer was measured and subtracted from the total absorbance, measured when corona-coated liposomes were mixed with the BCA reagent. Lipid concentration was quantified by Stewart assay, and Pb values (μg of protein/(μmol of lipid)) were then calculated.

### Mass Spectrometry

In-gel digestion of corona proteins (40 μg) was performed prior to LC-MS/MS analysis, as we have previously described.^23,27^ Digested samples were analyzed by LC-MS/MS using an UltiMate 3000 Rapid Separation LC (RSLC, Dionex Corp., Sunnyvale, CA, USA) coupled to a Q Exactive Hybrid Quadrupole-Orbitrap (Thermo Fisher Scientific, Waltham, MA, USA) mass spectrometer.

### Mass Spectrometry Data Analysis

To statistically compare the abundance of proteins identified in the liposomal coronas formed in APP/PS1 and WT mice, MS peak intensities were analyzed using Progenesis LC-MS software (v. 3.0; Nonlinear Dynamics). RAW files were imported into Progenesis LC-MS software (v. 3.0; Nonlinear Dynamics) with automatic feature detection enabled. A representative reference run was selected automatically, to which all other runs were aligned in a pairwise manner. Automatic processing was selected to run with applied filters for peaks charge state (maximum charge 5), and protein quantitation method, the relative quantitation using Hi-*N* with *N* = 3 peptides to measure per protein. The resulting MS/MS peak lists were exported as a single Mascot generic file and loaded onto a local Mascot Server (v. 2.3.0; Matrix Science, U.K.). The spectra were searched against the UniProt database using the following parameters: tryptic enzyme digestion with one missed cleavage allowed, peptide charges of +2 and +3, precursor mass tolerance of 15 mmu, fragment mass tolerance of 8 ppm, oxidation of methionines as variable modifications, and carbamidomethyl as fixed modifications, with decoy database search disabled and ESI-QUAD-TOF the selected instrument. Each search produced an XML file from Mascot, and the resulted peptides (XML files) were imported back into Progenesis LC-MS to assign peptides to features. Data were filtered to present a 1% false discovery rate (FDR) and a score above 21 through the “refine identification” tab of Progenesis QI toolbox. The resulting peptides were exported as an XML file from Mascot and imported back into Progenesis LC-MS to assign peptides to features.

### Pathway Enrichment Analysis

Proteins were classified by Kyoto Encyclopaedia of Genes & Genomes database using the Enrichr analysis tool. The pathway overlap network was constructed using the Gephi visualization platform. For each enriched pathway (network nodes) we calculated the proportion of constituent proteins that are shared with all other pathways (network edges). We then visualized all connections between pathways sharing more than a third of their constituent proteins. Pathway node size was determined by the number of constituent proteins in each pathway, and network edge thickness was determined by the proportion of shared proteins between the two connected pathways.

### Statistical Analysis

The effects of genotype on memory performance and protein abundance extracted were evaluated with Welch corrected *t* tests at each time point. Homoscedasticity and normality were evaluated graphically using predicted *vs* (Pearson) residuals and *Q*–*Q* plots. Plaque burden was analyzed by two-way ANOVA followed by Sidak corrected *post hoc* analyses. Statistical analyses of the data were performed using GraphPad Prism software. Accepted levels of significance were * *p* < 0.05, ** *p* < 0.01, and *** *p* < 0.001.

## Supporting Information Available

Figures S1 and S2 showing TEM images and longitudinal fluctuations and Tables S1–S6 listing blood proteins and results of molecular pathway enrichment analysis (PDF)
