# Supplementary material for: Nanoparticle-Enabled Enrichment of Longitudinal Blood Proteomic Fingerprints in Alzheimer’s Disease
Source: ACS Nano. 2021 Mar 17;15(4):7357–69. doi: 10.1021/acsnano.1c00658 (PMC8155389; doi:10.1021/acsnano.1c00658)
Supplement: Supplementary file 1 — nn1c00658_si_001.pdf [file nn1c00658_si_001.pdf]

SUPPORTING INFORMATION

Nanoparticle-Enabled Enrichment of Longitudinal Blood Proteomic Fingerprints in Alzheimer's Disease

Marilena Hadjidemetriou[1]*, Jack Rivers-Auty[2], Lana Papafilippou[1], James Eales[3], Katherine A.B. Kellett[2], Nigel M. Hooper[2], Catherine B. Lawrence[2], Kostas Kostarelos[1]*

[1]Nanomedicine Lab, School of Health Sciences, Faculty of Biology, Medicine and Health, The University of Manchester, Manchester M13 9PT, UK

[2]Division of Neuroscience and Experimental Psychology, School of Biological Sciences, Faculty of Biology, Medicine and Health, The University of Manchester, Manchester Academic Health Science Centre, Manchester M13 9PT, UK

[3]Division of Cardiovascular Sciences, School of Medical Sciences, Faculty of Biology, Medicine and Health, The University of Manchester M13 9PT, Manchester, UK

* Correspondence should be addressed to:
mairlena.hadjidemetriou@manchester.ac.uk and kostas.kostarelos@manchester.ac.uk

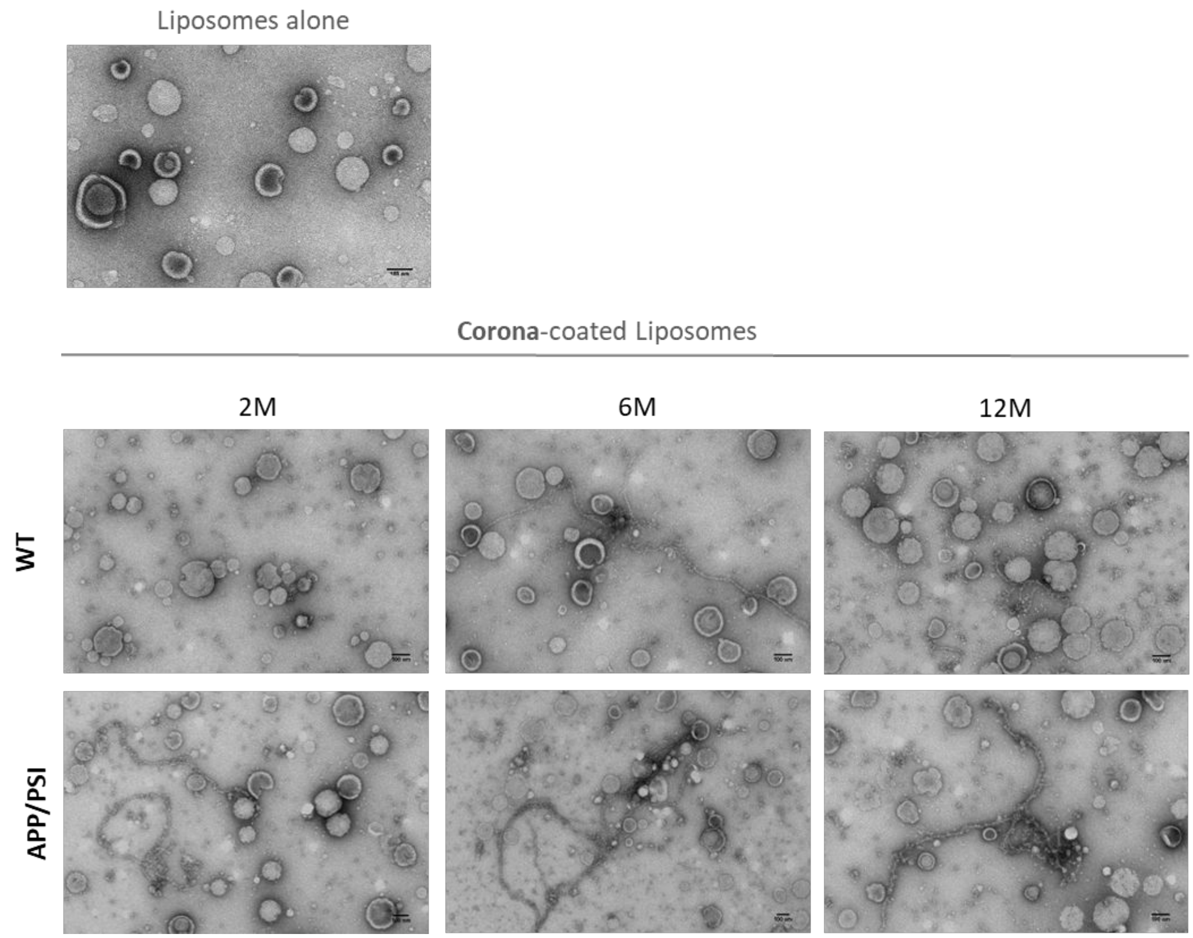

Figure S1: Negative stain TEM of liposomes before and after intravenous administration and recovery from the blood circulation of APP/PS1 and WT (C57/B6j) mice at 2, 6 and 12 months of age. All scale bars are 100nm.

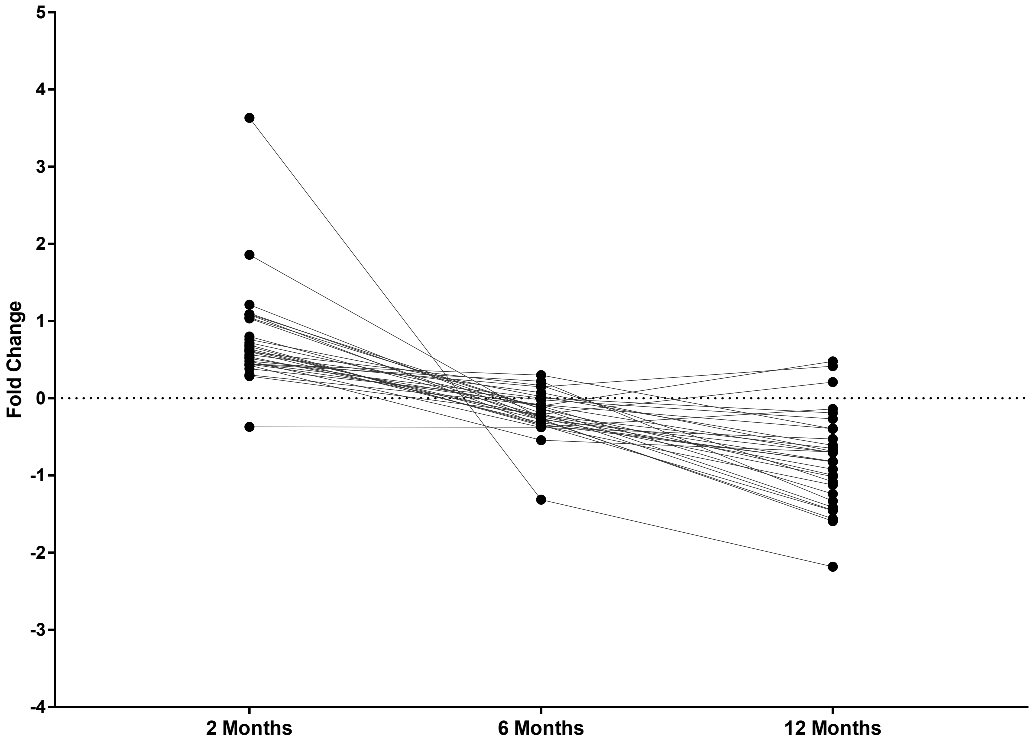

Figure S2: Longitudinal fluctuation in the fold change values of the **n=31** common proteins that were found to be differentially abundant between APP/PS1 and WT mice in both 2M and 12M time points.

Table S1: Full list of blood proteins identified by Progenesis analysis to be differentially abundant between **2-months** old APP/PS1 and WT mice. Only proteins with *p<0.05* are shown.

Description	Accession	Anova (p value)	Max fold change
**UPREGULATED (n= 101)**			
Pancreatic alpha-amylase OS=Mus musculus GN=Amy2 PE=1 SV=2	AMYP	5.71E-04	Infinity
Rab GDP dissociation inhibitor (Fragment) OS=Mus musculus GN=Gdi2 PE=1 SV=1	A0A1Y7VLG4	9.36E-04	4295.45
Monocarboxylate transporter 1 OS=Mus musculus GN=Slc16a1 PE=1 SV=1	MOT1	1.35E-03	72.28
Protein piccolo OS=Mus musculus GN=Pclo PE=1 SV=4	PCLO	5.56E-03	28.03
Solute carrier family 29 (Nucleoside transporters), member 1, isoform CRA_a OS=Mus musculus GN=Slc29a1 PE=2 SV=1	Q3TCZ2	2.97E-02	16.35
Protein KRI1 homolog OS=Mus musculus GN=Kri1 PE=1 SV=3	KRI1	4.18E-02	12.65
Basigin OS=Mus musculus GN=Bsg PE=1 SV=2	BASI	1.59E-02	12.28
Early endosome antigen 1 OS=Mus musculus GN=Eea1 PE=1 SV=2	EEA1	5.06E-03	12.21
Uncharacterized protein OS=Mus musculus GN=Cd36 PE=2 SV=1	Q3TA14	3.85E-02	12.10
Cytochrome b5 OS=Mus musculus GN=Cyb5a PE=1 SV=2	CYB5	6.52E-03	11.19
Sarcoplasmic/endoplasmic reticulum calcium ATPase 2 OS=Mus musculus GN=Atp2a2 PE=1 SV=2	AT2A2	1.25E-02	11.12
Disintegrin and metalloproteinase domain-containing protein 10 OS=Mus musculus GN=Adam10 PE=1 SV=2	ADA10	2.81E-02	10.81
T-complex protein 1 subunit alpha OS=Mus musculus GN=Tcp1 PE=1 SV=3	TCPA	1.27E-02	9.35
60S ribosomal protein L18 OS=Mus musculus GN=Rpl18 PE=1 SV=1	A0A1B0GQU8	1.42E-02	7.69
Receptor expression-enhancing protein OS=Mus musculus GN=Reep6 PE=1 SV=1	D3Z6V2	3.06E-02	7.44
E3 ubiquitin-protein ligase UBR4 OS=Mus musculus GN=Ubr4 PE=1 SV=1	UBR4	1.11E-03	6.98
Uncharacterized protein OS=Mus musculus GN=Psmc5 PE=2 SV=1	A0A1S6GWH1	4.72E-02	6.85
26S proteasome non-ATPase regulatory subunit 1 OS=Mus musculus GN=Psmd1 PE=1 SV=1	PSMD1	7.99E-03	6.57
Filamin-A OS=Mus musculus GN=Flna PE=1 SV=5	FLNA	1.99E-03	6.33
Integrin beta OS=Mus musculus GN=Itgb2 PE=1 SV=1	M0QWA7	2.71E-03	5.99
Predicted gene 45713 OS=Mus musculus GN=Gm45713 PE=2 SV=1	A0A1B0GS68	1.86E-02	5.88
ATP synthase subunit beta, mitochondrial OS=Mus musculus GN=Atp5b PE=1 SV=2	ATPB	3.90E-02	5.78
Ribosomal protein L15 OS=Mus musculus GN=Rpl15 PE=2 SV=1	Q3U7D2	4.80E-02	5.58
26S proteasome regulatory subunit 6A (Fragment) OS=Mus musculus GN=Psmc3 PE=1 SV=6	A0A087WPH7	3.87E-02	5.46
Uncharacterized protein OS=Mus musculus GN=Rpl14 PE=2 SV=1	Q9CWK0	4.59E-02	5.42
Long-chain-fatty-acid--CoA ligase 5 OS=Mus musculus GN=Acsl5 PE=1 SV=1	ACSL5	3.30E-02	5.41
Ribosomal protein S2, pseudogene 6 OS=Mus musculus GN=Rps2-ps6 PE=3 SV=1	A0A140T8L5	2.25E-02	5.28
Kell blood group glycoprotein homolog OS=Mus musculus GN=Kel PE=1 SV=1	KELL	2.69E-02	5.24
Calreticulin OS=Mus musculus GN=Calr PE=1 SV=1	CALR	1.19E-02	5.13
Erythrocyte band 7 integral membrane protein OS=Mus musculus GN=Stom PE=1 SV=3	STOM	2.40E-02	4.93
Moesin OS=Mus musculus GN=Msn PE=1 SV=3	MOES	6.20E-03	4.69
60S ribosomal protein L6 OS=Mus musculus GN=Rpl6 PE=1 SV=1	Q3UCH0	1.70E-02	4.64
26S proteasome regulatory subunit 10B OS=Mus musculus GN=Psmc6 PE=1 SV=1	PRS10	3.22E-02	4.49
Talin-1 OS=Mus musculus GN=Tln1 PE=1 SV=2	TLN1	1.48E-02	4.40

RAS-related C3 botulinum substrate 3, isoform CRA_a (Fragment) OS=Mus musculus GN=Rac3 PE=1 SV=1	A2AC13	8.87E-04	4.38
Laminin subunit alpha-2 OS=Mus musculus GN=Lama2 PE=1 SV=2	LAMA2	7.84E-03	4.26
IgM heavy chain VDJ region (Fragment) OS=Mus musculus PE=2 SV=1	X5J5A8	1.33E-03	4.19
Aquaporin-1 OS=Mus musculus GN=Aqp1 PE=1 SV=3	AQP1	1.93E-02	4.17
Uncharacterized protein OS=Mus musculus GN=Rpl7 PE=2 SV=1	Q3UBI6	2.28E-02	4.02
Annexin A5 OS=Mus musculus GN=Anxa5 PE=1 SV=1	ANXA5	4.51E-03	4.02
Glucosidase 2 subunit beta OS=Mus musculus GN=Prkcsh PE=1 SV=1	GLU2B	2.47E-03	3.97
VH region (Fragment) OS=Mus musculus PE=2 SV=1	Q53VQ0	2.83E-02	3.94
Clathrin heavy chain 1 OS=Mus musculus GN=Cltc PE=1 SV=3	CLH1	1.80E-02	3.68
Complement C1q tumor necrosis factor-related protein 3 OS=Mus musculus GN=C1qtnf3 PE=2 SV=1	C1QT3	3.68E-02	3.59
Proteoglycan 4 OS=Mus musculus GN=Prg4 PE=1 SV=1	A0A0R4J207	1.97E-02	3.47
Ezrin OS=Mus musculus GN=Ezr PE=1 SV=3	EZRI	6.84E-03	3.46
26S proteasome non-ATPase regulatory subunit 14 OS=Mus musculus GN=Psmd14 PE=1 SV=2	PSDE	4.20E-02	3.31
Immunoglobulin heavy variable 10-1 (Fragment) OS=Mus musculus GN=Ighv10-1 PE=4 SV=1	A0A0B4J1J6	3.80E-02	3.30
Ig kappa chain V-III region 50S10.1 OS=Mus musculus PE=1 SV=1	KV3A4	1.90E-02	3.29
GDH/6PGL endoplasmic bifunctional protein OS=Mus musculus GN=H6pd PE=1 SV=1	A2A7A7	1.57E-03	3.11
Ras-related protein Rap-1b OS=Mus musculus GN=Rap1b PE=1 SV=2	RAP1B	3.43E-02	3.02
Ig kappa chain V-III region PC 3741/TEPC 111 OS=Mus musculus PE=1 SV=1	KV3A8	4.38E-02	3.02
Endoplasmin OS=Mus musculus GN=Hsp90b1 PE=1 SV=2	ENPL	2.44E-03	3.02
WD repeat-containing protein 90 OS=Mus musculus GN=Wdr90 PE=1 SV=1	H7BX49	8.74E-03	2.99
Proteasome subunit beta type-5 OS=Mus musculus GN=Psmb5 PE=1 SV=3	PSB5	1.71E-02	2.90
Protein Igkv5-48 (Fragment) OS=Mus musculus GN=Igkv5-48 PE=1 SV=2	A0A140T8N2	5.51E-04	2.83
Immunoglobulin lambda variable 3 (Fragment) OS=Mus musculus GN=Iglv3 PE=4 SV=1	A0A0B4J1K5	1.75E-02	2.79
Spectrin alpha chain, non-erythrocytic 1 OS=Mus musculus GN=Sptan1 PE=1 SV=4	SPTN1	4.22E-02	2.77
MCG23377, isoform CRA_b OS=Mus musculus GN=Gm8797 PE=4 SV=1	A0A0A6YW67	1.64E-02	2.76
Aminopeptidase N (Fragment) OS=Mus musculus GN=Anpep PE=1 SV=1	A0A0U1RNS3	4.31E-02	2.69
Proteasome subunit beta type-6 OS=Mus musculus GN=Psmb6 PE=1 SV=3	PSB6	1.20E-03	2.68
40S ribosomal protein S9 OS=Mus musculus GN=Rps9 PE=1 SV=1	D3YWH9	4.57E-02	2.60
Elongation factor 1-alpha 1 OS=Mus musculus GN=Eef1a1 PE=1 SV=3	EF1A1	1.39E-02	2.57
Myosin-9 OS=Mus musculus GN=Myh9 PE=1 SV=4	MYH9	2.98E-02	2.57
Proteasome subunit beta type-4 OS=Mus musculus GN=Psmb4 PE=1 SV=1	PSB4	1.33E-03	2.56
Immunoglobulin kappa chain variable 1-122 (Fragment) OS=Mus musculus GN=Igkv1-122 PE=4 SV=2	A0A140T8M9	3.35E-02	2.50
Retinol dehydrogenase 7 OS=Mus musculus GN=Rdh7 PE=1 SV=1	RDH7	2.08E-02	2.48
Immunoglobulin kappa chain variable 9-124 OS=Mus musculus GN=Igkv9-124 PE=1 SV=7	A0A075B5K2	2.72E-02	2.47
Uncharacterized protein OS=Mus musculus GN=Hspa5 PE=2 SV=1	Q3TI47	9.21E-03	2.41
Uncharacterized protein OS=Mus musculus GN=Thbs1 PE=2 SV=1	Q3TR40	4.13E-02	2.36
Ig kappa chain V-V region MOPC 149 OS=Mus musculus PE=1 SV=1	KV5A4	3.72E-02	2.35
Uroplakin-3b OS=Mus musculus GN=Upk3b PE=1 SV=1	A0A0R4J0S8	1.98E-02	2.32
Immunoglobulin heavy variable V1-11 (Fragment) OS=Mus musculus GN=Ighv1-11 PE=4 SV=1	A0A0A6YWI9	1.97E-02	2.30
Immunoglobulin kappa variable 12-44 (Fragment) OS=Mus musculus GN=Igkv12-44 PE=4 SV=2	A0A140T8M2	1.41E-02	2.27
Immunoglobulin kappa variable 8-28 OS=Mus musculus GN=Igkv8-28 PE=1 SV=7	A0A075B5N3	2.31E-02	2.27

Tankyrase-2 OS=Mus musculus GN=Tnks2 PE=2 SV=2	TNKS2	6.45E-03	2.23
Cartilage oligomeric matrix protein OS=Mus musculus GN=Comp PE=1 SV=2	COMP	2.39E-02	2.22
Immunoglobulin kappa chain variable 8-27 OS=Mus musculus GN=Igkv8-27 PE=4 SV=1	A0A075B5N4	4.33E-02	2.17
Proteasome subunit alpha type-6 OS=Mus musculus GN=Psma6 PE=1 SV=1	PSA6	9.93E-03	2.08
Proteasome subunit alpha type (Fragment) OS=Mus musculus GN=Psma4 PE=2 SV=1	Q3TL95	4.15E-02	2.05
Immunoglobulin kappa variable 6-17 (Fragment) OS=Mus musculus GN=Igkv6-17 PE=1 SV=2	A0A140T8Q3	4.37E-02	2.01
Integrin beta-1 OS=Mus musculus GN=Itgb1 PE=1 SV=1	ITB1	1.20E-02	2.01
Immunoglobulin kappa variable 2-109 (Fragment) OS=Mus musculus GN=Igkv2-109 PE=4 SV=4	A0A075B5K6	6.31E-03	2.00
Immunoglobulin heavy variable V1-59 OS=Mus musculus GN=Ighv1-59 PE=4 SV=1	A0A075B5X0	1.04E-03	1.99
Immunoglobulin kappa variable 4-53 OS=Mus musculus GN=Igkv4-53 PE=1 SV=1	A0A075B677	2.98E-02	1.99
Coagulation factor V OS=Mus musculus GN=F5 PE=1 SV=1	FA5	2.48E-03	1.99
Single-chain variable OS=Mus musculus GN=Wgn-scFv PE=2 SV=1	A0A1E1GJG6	2.64E-02	1.98
DNA helicase B OS=Mus musculus GN=Helb PE=1 SV=2	HELB	1.46E-03	1.98
Immunoglobulin kappa variable 6-23 (Fragment) OS=Mus musculus GN=Igkv6-23 PE=4 SV=2	A0A140T8N5	4.94E-03	1.94
Napsin-A OS=Mus musculus GN=Napsa PE=1 SV=1	NAPSA	2.49E-02	1.92
Aberrantly recombined kappa chain Vk8/J1 region (Fragment) OS=Mus musculus PE=1 SV=1	A2N494	4.19E-02	1.90
Light chain variable region (Fragment) OS=Mus musculus PE=2 SV=1	A2NVX0	1.11E-02	1.87
Adiponectin OS=Mus musculus GN=Adipoq PE=1 SV=2	ADIPO	3.69E-02	1.83
Long-chain-fatty-acid--CoA ligase 1 OS=Mus musculus GN=Acsl1 PE=1 SV=2	ACSL1	1.60E-02	1.81
Transferrin receptor protein 1 OS=Mus musculus GN=Tfrc PE=1 SV=1	TFR1	2.17E-04	1.81
Anti-VIPase light chain variable region (Fragment) OS=Mus musculus PE=4 SV=1	Q8K1F1	3.96E-02	1.78
Maltase-glucoamylase OS=Mus musculus GN=Mgam PE=1 SV=1	B5THE2	5.14E-03	1.74
Proteasome subunit alpha type-3 OS=Mus musculus GN=Psma3 PE=1 SV=3	PSA3	4.56E-02	1.70
Anti-myosin immunoglobulin light chain variable region (Fragment) OS=Mus musculus PE=2 SV=1	Q9JL74	3.95E-02	1.66
Argininosuccinate lyase OS=Mus musculus GN=Asl PE=1 SV=1	ARLY	2.48E-02	1.42
Clusterin OS=Mus musculus GN=Clu PE=1 SV=1	CLUS	2.51E-02	1.27
**DOWNREGULATED (n=4)**			
IgG1 heavy chain VDJ region (Fragment) OS=Mus musculus PE=2 SV=1	X5J5A8	1.83E-02	9.03
Colony stimulating factor 1 receptor OS=Mus musculus GN=Csf1r PE=1 SV=1	Q0P0S6	4.66E-02	3.82
Fibronectin OS=Mus musculus GN=Fn1 PE=1 SV=1	A0A087WR50	2.56E-02	2.35
Serum amyloid A-4 protein OS=Mus musculus GN=Saa4 PE=1 SV=2	SAA4	4.57E-02	1.69

Table S2: Full list of blood proteins identified by Progenesis analysis to be differentially abundant between **6-months** old APP/PS1 and WT mice. Only proteins with *p<0.05* are shown.

Description	Accession	Anova (p value)	Max fold change
**UPREGULATED (n= 19)**			
Immunoglobulin heavy variable V1-43 OS=Mus musculus GN=Ighv1-43 PE=4 SV=1	A0A075B5V7	5.23E-03	1.98
Filamin-B OS=Mus musculus GN=Flnb PE=1 SV=3	FLNB	5.24E-03	31.55
Argininosuccinate synthase OS=Mus musculus GN=Ass1 PE=1 SV=1	ASSY	6.54E-03	4.28
Pyrethroid hydrolase Ces2e OS=Mus musculus GN=Ces2e PE=1 SV=1	EST2E	8.64E-03	1.75
Uroplakin-3b OS=Mus musculus GN=Upk3b PE=1 SV=1	A0A0R4J0S8	1.07E-02	1.75
Major urinary protein 10 OS=Mus musculus GN=Mup10 PE=2 SV=1	A2BIN1	1.29E-02	3.66
Napsin-A OS=Mus musculus GN=Napsa PE=1 SV=1	NAPSA	1.72E-02	1.47
Retinol dehydrogenase 7 OS=Mus musculus GN=Rdh7 PE=1 SV=1	RDH7	2.57E-02	2.65
Thyrotropin-releasing hormone-degrading ectoenzyme OS=Mus musculus GN=Trhde PE=1 SV=1	TRHDE	2.85E-02	3.68
Meprin A subunit OS=Mus musculus GN=Mep1a PE=1 SV=1	A0A0R4J043	2.88E-02	3.43
Uncharacterized protein OS=Mus musculus GN=Thbs1 PE=2 SV=1	Q3TR40	3.14E-02	2.58
Tankyrase-2 OS=Mus musculus GN=Tnks2 PE=2 SV=2	TNKS2	3.16E-02	2.04
Complement C1q tumor necrosis factor-related protein 3 OS=Mus musculus GN=C1qtnf3 PE=2 SV=1	C1QT3	3.55E-02	1.77
Cytosolic 10-formyltetrahydrofolate dehydrogenase OS=Mus musculus GN=Aldh1l1 PE=1 SV=1	AL1L1	3.96E-02	5.13
G protein-coupled receptor 179 OS=Mus musculus GN=Gpr179 PE=4 SV=1	E9PY61	4.08E-02	1.32
Thrombospondin-4 OS=Mus musculus GN=Thbs4 PE=1 SV=1	TSP4	4.49E-02	1.49
C-type lectin domain family 11 member A OS=Mus musculus GN=Clec11a PE=1 SV=1	CLC11	4.82E-02	2.69
MCG5400 OS=Mus musculus GN=Myl12a PE=1 SV=1	Q6ZWQ9	4.92E-02	4.31
**DOWNREGULATED (n=11)**			
CD5 antigen-like OS=Mus musculus GN=Cd5l PE=1 SV=3	CD5L	1.23E-03	2.65
Immunoglobulin heavy variable V12-3 (Fragment) OS=Mus musculus GN=Ighv12-3 PE=4 SV=2	A0A075B5T1	1.72E-03	3.24
Light chain variable region (Fragment) OS=Mus musculus PE=2 SV=1	A2NVX0	2.48E-03	1.77
Oncoprotein-induced transcript 3 protein OS=Mus musculus GN=Oit3 PE=2 SV=2	OIT3	3.38E-03	3.81
IgM heavy chain VDJ region (Fragment) OS=Mus musculus PE=2 SV=1	X5J4G2	5.94E-03	1.55
Bisphosphoglycerate mutase OS=Mus musculus GN=Bpgm PE=1 SV=2	PMGE	7.21E-03	14.62
Properdin OS=Mus musculus GN=Cfp PE=2 SV=2	PROP	2.11E-02	1.83
Anti-colorectal carcinoma light chain OS=Mus musculus PE=1 SV=1	Q7TS98	2.78E-02	2.70
Protein piccolo OS=Mus musculus GN=Pclo PE=1 SV=4	PCLO	2.80E-02	3.60
Immunoglobulin heavy variable 7-1 (Fragment) OS=Mus musculus GN=Ighv7-1 PE=1 SV=2	A0A075B5S2	4.82E-02	1.86
Immunoglobulin heavy variable 1-39 (Fragment) OS=Mus musculus GN=Ighv1-39 PE=4 SV=1	A0A075B5V5	4.98E-02	1.39

Table S3: Full list of blood proteins identified by Progenesis analysis to be differentially abundant between 1**2-months** old APP/PS1 and WT mice.

Description	Accession	Anova (p value)	Max fold change
**UPREGULATED (n= 8)**			
MRP5 (Fragment) OS=Mus musculus GN=Ighv9-4 PE=2 SV=1	Q925S1	7.28E-04	3.84
Ig kappa chain V-III region PC 3741/TEPC 111 OS=Mus musculus PE=1 SV=1	KV3A8	7.86E-04	2.61
Immunoglobulin kappa variable 4-80 (Fragment) OS=Mus musculus GN=Igkv4-80 PE=4 SV=2	A0A075B5L7	3.37E-03	2.49
Glycogen phosphorylase, muscle form OS=Mus musculus GN=Pygm PE=1 SV=3	PYGM	3.76E-02	6.42
Immunoglobulin heavy variable V9-3 (Fragment) OS=Mus musculus GN=Ighv9-3 PE=1 SV=1	A0A0B4J1J5	3.88E-02	1.35
Igh protein OS=Mus musculus GN=Igh PE=2 SV=1	Q4VAB6	3.95E-02	4.48
Ig kappa chain V-III region 50S10.1 OS=Mus musculus PE=1 SV=1	KV3A4	4.00E-02	3.00
Immunoglobulin kappa variable 6-23 (Fragment) OS=Mus musculus GN=Igkv6-23 PE=4 SV=2	A0A140T8N5	4.74E-02	1.62
**DOWNREGULATED (n= 97)**			
Caveolae-associated protein 1 OS=Mus musculus GN=Cavin1 PE=1 SV=1	CAVN1	4.42E-05	Infinity
Solute carrier family 2, facilitated glucose transporter member 3 OS=Mus musculus GN=Slc2a3 PE=1 SV=1	GTR3	5.69E-05	Infinity
Dihydrolipoyllysine-residue acetyltransferase component of pyruvate dehydrogenase complex, mitochondrial OS=Mus musculus GN=Dlat PE=1 SV=2	ODP2	1.64E-04	14.72
Bile acyl-CoA synthetase OS=Mus musculus GN=Slc27a5 PE=1 SV=1	E9PXV4	4.41E-04	Infinity
NADPH--cytochrome P450 reductase (Fragment) OS=Mus musculus GN=Por PE=1 SV=1	E9PVT9	8.02E-04	Infinity
Uncharacterized protein OS=Mus musculus GN=Epb42 PE=2 SV=1	Q3T9M7	8.63E-04	2.23
Uncharacterized protein OS=Mus musculus GN=Cd36 PE=2 SV=1	Q3TA14	1.76E-03	28.50
Erythrocyte band 7 integral membrane protein OS=Mus musculus GN=Stom PE=1 SV=3	STOM	1.95E-03	13.27
Sarcoplasmic/endoplasmic reticulum calcium ATPase 2 OS=Mus musculus GN=Atp2a2 PE=1 SV=2	AT2A2	3.13E-03	9.93
Immunoglobulin lambda variable 3 (Fragment) OS=Mus musculus GN=Iglv3 PE=4 SV=1	A0A0B4J1K5	3.30E-03	2.48
Fibronectin OS=Mus musculus GN=Fn1 PE=1 SV=1	A0A087WR50	3.64E-03	1.38
Moesin OS=Mus musculus GN=Msn PE=1 SV=3	MOES	4.90E-03	6.57
Tripeptidyl-peptidase 2 OS=Mus musculus GN=Tpp2 PE=1 SV=3	TPP2	5.41E-03	2.25
Kell blood group glycoprotein homolog OS=Mus musculus GN=Kel PE=1 SV=1	KELL	5.49E-03	17.34
Uncharacterized protein OS=Mus musculus GN=Uba1 PE=2 SV=1	A0A1S6GWH5	6.74E-03	2.34
Ezrin OS=Mus musculus GN=Ezr PE=1 SV=3	EZRI	6.87E-03	6.60
RAS-related C3 botulinum substrate 3, isoform CRA_a (Fragment) OS=Mus musculus GN=Rac3 PE=1 SV=1	A2AC13	6.89E-03	5.02
Solute carrier family 29 (Nucleoside transporters), member 1, isoform CRA_a OS=Mus musculus GN=Slc29a1 PE=2 SV=1	Q3TCZ2	7.27E-03	39.06
Peroxiredoxin-2 OS=Mus musculus GN=Prdx2 PE=1 SV=3	PRDX2	7.94E-03	2.59
Immunoglobulin heavy variable 11-1 (Fragment) OS=Mus musculus GN=Ighv11-1 PE=4 SV=1	A0A075B5R6	7.98E-03	3.33
Disintegrin and metalloproteinase domain-containing protein 10 OS=Mus musculus GN=Adam10 PE=1 SV=2	ADA10	1.00E-02	36.31
Ras-related protein Rap-1b OS=Mus musculus GN=Rap1b PE=1 SV=2	RAP1B	1.02E-02	3.36
ATP synthase subunit beta, mitochondrial OS=Mus musculus GN=Atp5b PE=1 SV=2	ATPB	1.07E-02	4.06
Heat shock 70 kDa protein 4 OS=Mus musculus GN=Hspa4 PE=1 SV=1	Q3U2G2	1.16E-02	2.98

Phospholipid transfer protein OS=Mus musculus GN=Pltp PE=1 SV=1	PLTP	1.23E-02	1.61
Integrin beta-3 OS=Mus musculus GN=Itgb3 PE=1 SV=2	ITB3	1.29E-02	9.77
Aquaporin-1 OS=Mus musculus GN=Aqp1 PE=1 SV=3	AQP1	1.44E-02	8.30
Annexin A5 OS=Mus musculus GN=Anxa5 PE=1 SV=1	ANXA5	1.48E-02	6.64
Alpha actinin 1a OS=Mus musculus GN=Actn1 PE=1 SV=1	A1BN54	1.53E-02	6.43
EMILIN-1 OS=Mus musculus GN=Emilin1 PE=1 SV=1	EMIL1	1.53E-02	11.70
Spectrin alpha chain, non-erythrocytic 1 OS=Mus musculus GN=Sptan1 PE=1 SV=4	SPTN1	1.54E-02	21.44
Large neutral amino acids transporter small subunit 3 OS=Mus musculus GN=Slc43a1 PE=1 SV=1	A2ATS4	1.63E-02	202.52
Integrin alpha 2b OS=Mus musculus GN=Itga2b PE=2 SV=1	B2RPR7	1.63E-02	6.66
Basigin OS=Mus musculus GN=Bsg PE=1 SV=2	BASI	1.64E-02	25.79
Angiopoietin-1 OS=Mus musculus GN=Angpt1 PE=1 SV=2	ANGP1	1.66E-02	6.42
Glyceraldehyde-3-phosphate dehydrogenase OS=Mus musculus GN=Gapdh PE=1 SV=1	A0A0A0MQF6	1.71E-02	2.31
Maltase-glucoamylase OS=Mus musculus GN=Mgam PE=1 SV=1	A0A0J9YVE6	1.73E-02	1.20
Perilipin-4 OS=Mus musculus GN=Plin4 PE=1 SV=2	PLIN4	1.76E-02	64.24
Uncharacterized protein OS=Mus musculus PE=2 SV=1	I6L9E1	1.83E-02	2.22
Uncharacterized protein (Fragment) OS=Mus musculus GN=Tpp2 PE=2 SV=1	Q3TB11	1.91E-02	9.38
Microsomal triglyceride transfer protein large subunit OS=Mus musculus GN=Mttp PE=1 SV=2	MTP	1.95E-02	25.10
Bifunctional purine biosynthesis protein PURH OS=Mus musculus GN=Atic PE=1 SV=2	PUR9	1.95E-02	5.85
Neuronal membrane glycoprotein M6-a OS=Mus musculus GN=Gpm6a PE=1 SV=1	GPM6A	2.09E-02	14.99
Monocarboxylate transporter 1 OS=Mus musculus GN=Slc16a1 PE=1 SV=1	MOT1	2.20E-02	10.32
Guanine nucleotide-binding protein G(I)/G(S)/G(T) subunit beta-1 OS=Mus musculus GN=Gnb1 PE=1 SV=3	GBB1	2.27E-02	5.66
Glucosidase 2 subunit beta OS=Mus musculus GN=Prkcsh PE=1 SV=1	GLU2B	2.28E-02	12.09
Hsp90aa1 protein (Fragment) OS=Mus musculus GN=Hsp90aa1 PE=2 SV=1	A0PJ91	2.32E-02	2.02
ATP synthase subunit alpha, mitochondrial OS=Mus musculus GN=Atp5a1 PE=1 SV=1	ATPA	2.38E-02	15.24
Dimethylaniline monooxygenase [N-oxide-forming] 5 OS=Mus musculus GN=Fmo5 PE=1 SV=4	FMO5	2.39E-02	9.17
Coronin OS=Mus musculus GN=Coro1a PE=2 SV=1	Q3T9L1	2.39E-02	4.04
Integrin-linked protein kinase OS=Mus musculus GN=Ilk PE=1 SV=2	ILK	2.42E-02	3.28
Galectin-3-binding protein OS=Mus musculus GN=Lgals3bp PE=1 SV=1	LG3BP	2.46E-02	5.45
Spectrin beta chain, erythrocytic OS=Mus musculus GN=Sptb PE=1 SV=4	SPTB1	2.53E-02	4.93
RAS oncogene family protein OS=Mus musculus GN=Rab11b PE=2 SV=1	A0A068BFR3	2.63E-02	3.58
Rab GDP dissociation inhibitor (Fragment) OS=Mus musculus GN=Gdi2 PE=1 SV=1	A0A1Y7VLG4	2.64E-02	151.96
Dipeptidyl peptidase 4 OS=Mus musculus GN=Dpp4 PE=1 SV=3	DPP4	2.67E-02	10.43
Filamin-A OS=Mus musculus GN=Flna PE=1 SV=5	FLNA	2.77E-02	4.44
MCG23377, isoform CRA_b OS=Mus musculus GN=Gm8797 PE=4 SV=1	A0A0A6YW67	2.81E-02	2.47
Clathrin heavy chain 1 OS=Mus musculus GN=Cltc PE=1 SV=3	CLH1	2.83E-02	4.86
Adenylyl cyclase-associated protein 1 OS=Mus musculus GN=Cap1 PE=1 SV=4	CAP1	2.84E-02	2.13
Annexin A2 OS=Mus musculus GN=Anxa2 PE=1 SV=2	ANXA2	2.85E-02	7.47
CD9 antigen OS=Mus musculus GN=Cd9 PE=1 SV=2	CD9	2.85E-02	3.11
Tubulin beta-4A chain OS=Mus musculus GN=Tubb4a PE=1 SV=3	TBB4A	2.86E-02	3.28
Spectrin beta chain OS=Mus musculus GN=Sptb PE=2 SV=1	B2RX08	2.88E-02	2.91
Lactadherin OS=Mus musculus GN=Mfge8 PE=1 SV=3	MFGM	2.99E-02	4.26
Calnexin OS=Mus musculus GN=Canx PE=1 SV=1	CALX	3.10E-02	37.72

26S proteasome non-ATPase regulatory subunit 2 OS=Mus musculus GN=Psmd2 PE=1 SV=1	PSMD2	3.29E-02	2.24
UDP-glucuronosyltransferase 1-1 OS=Mus musculus GN=Ugt1a1 PE=1 SV=2	UD11	3.47E-02	9.57
Decorin OS=Mus musculus GN=Dcn PE=1 SV=1	PGS2	3.48E-02	5.23
Leucine-rich repeat neuronal protein 4 OS=Mus musculus GN=Lrrn4 PE=1 SV=2	LRRN4	3.53E-02	6.74
Annexin A6 OS=Mus musculus GN=Anxa6 PE=1 SV=3	ANXA6	3.65E-02	80.55
Mannan-binding lectin serine protease 2 OS=Mus musculus GN=Masp2 PE=1 SV=1	MASP2	3.68E-02	1.50
Filamin-B OS=Mus musculus GN=Flnb PE=1 SV=3	FLNB	3.74E-02	5.37
Integrin beta-1 OS=Mus musculus GN=Itgb1 PE=1 SV=1	ITB1	4.00E-02	5.08
Immunoglobulin kappa variable 8-34 (Fragment) OS=Mus musculus GN=Igkv8-34 PE=4 SV=4	A0A0G2JDU0	4.03E-02	15.10
Aminopeptidase N (Fragment) OS=Mus musculus GN=Anpep PE=1 SV=1	A0A0U1RNS3	4.04E-02	4.95
55 kDa erythrocyte membrane protein OS=Mus musculus GN=Mpp1 PE=1 SV=1	B7ZCL8	4.07E-02	46.48
Dihydrolipoyllysine-residue succinyltransferase component of 2-oxoglutarate dehydrogenase complex, mitochondrial OS=Mus musculus GN=Dlst PE=1 SV=1	ODO2	4.12E-02	2.62
Transketolase (Fragment) OS=Mus musculus GN=Tkt PE=1 SV=1	A0A286YE28	4.15E-02	2.85
Protein Igkv5-48 (Fragment) OS=Mus musculus GN=Igkv5-48 PE=1 SV=2	A0A140T8N2	4.17E-02	1.54
Serglycin OS=Mus musculus GN=Srgn PE=1 SV=1	SRGN	4.34E-02	7.41
Laminin subunit gamma-1 OS=Mus musculus GN=Lamc1 PE=1 SV=1	F8VQJ3	4.41E-02	2.26
Anti-myosin immunoglobulin light chain variable region (Fragment) OS=Mus musculus GN=Igkv4-91 PE=2 SV=1	Q9JL78	4.49E-02	3.51
Uncharacterized protein OS=Mus musculus GN=Cyp2c37 PE=2 SV=1	Q9DBD9	4.50E-02	19.04
Mannan-binding lectin serine protease 1 OS=Mus musculus GN=Masp1 PE=1 SV=2	MASP1	4.56E-02	1.35
Vinculin OS=Mus musculus GN=Vcl PE=1 SV=4	VINC	4.57E-02	2.39
Ankyrin-1 OS=Mus musculus GN=Ank1 PE=1 SV=1	A0A0R4J1N7	4.60E-02	5.50
Protein KRI1 homolog OS=Mus musculus GN=Kri1 PE=1 SV=3	KRI1	4.61E-02	28.14
UTP--glucose-1-phosphate uridylyltransferase OS=Mus musculus GN=Ugp2 PE=1 SV=3	UGPA	4.66E-02	3.26
Fermitin family homolog 3 OS=Mus musculus GN=Fermt3 PE=1 SV=1	URP2	4.70E-02	1.78
Retinal dehydrogenase 1 OS=Mus musculus GN=Aldh1a1 PE=1 SV=5	AL1A1	4.73E-02	2.46
Laminin subunit gamma-1 OS=Mus musculus GN=Lamc1 PE=1 SV=2	LAMC1	4.81E-02	3.21
Serum paraoxonase/arylesterase 1 OS=Mus musculus GN=Pon1 PE=1 SV=2	PON1	4.92E-02	1.59
L-lactate dehydrogenase OS=Mus musculus GN=Ldha PE=1 SV=1	A0A1B0GSR9	4.92E-02	1.57
Uncharacterized protein OS=Mus musculus GN=Hspa5 PE=2 SV=1	Q3TI47	4.93E-02	1.85
ADP/ATP translocase 2 OS=Mus musculus GN=Slc25a5 PE=1 SV=3	ADT2	4.95E-02	7.18
Annexin OS=Mus musculus GN=Anxa7 PE=1 SV=1	A0A2C9F2D2	4.98E-02	10.61

Table S4: Full list of blood proteins identified by Progenesis analysis to be differentially abundant between **2- and 6-months** old APP/PS1 mice.

Description	Accession	Anova (p value)	Max fold change
**UPREGULATED (n= 10)**			
HC protein OS=Mus musculus GN=HC PE=2 SV=1	A0A0C6E3V3	8.39E-06	Infinity
Immunoglobulin heavy variable V1-43 OS=Mus musculus GN=Ighv1-43 PE=4 SV=1	A0A075B5V7	9.00E-06	2.12
Thyroxine-binding globulin OS=Mus musculus GN=Serpina7 PE=2 SV=1	THBG	2.18E-04	Infinity
Uncharacterized protein OS=Mus musculus PE=2 SV=1	Q2TAW9	2.42E-03	17.22
Complement component C8 alpha chain OS=Mus musculus GN=C8a PE=1 SV=1	CO8A	1.14E-02	3.60
IgG1 heavy chain VDJ region (Fragment) OS=Mus musculus PE=2 SV=1	X5J4T6	1.78E-02	46.85
Serum amyloid A-4 protein OS=Mus musculus GN=Saa4 PE=1 SV=2	SAA4	2.24E-02	1.94
Uncharacterized protein (Fragment) OS=Mus musculus GN=Rbp4 PE=2 SV=1	Q3TF08	3.03E-02	5.85
Phospholipid transfer protein OS=Mus musculus GN=Pltp PE=1 SV=1	PLTP	3.91E-02	1.95
IgA heavy chain variable region (Fragment) OS=Mus musculus PE=2 SV=1	K7THF9	4.13E-02	19.88
**DOWNREGULATED (n= 65)**			
Immunoglobulin heavy variable V1-11 (Fragment) OS=Mus musculus GN=Ighv1-11 PE=4 SV=1	A0A0A6YWI9	3.57E-02	2.71
Uncharacterized protein OS=Mus musculus GN=Serinc3 PE=2 SV=1	Q3TWZ3	3.63E-02	4.22
Uncharacterized protein OS=Mus musculus GN=Hspa5 PE=2 SV=1	Q3TI47	3.64E-02	2.40
Immunoglobulin kappa variable 8-34 (Fragment) OS=Mus musculus GN=Igkv8-34 PE=4 SV=4	A0A0G2JDU0	3.69E-02	9.15
14-3-3 protein zeta/delta OS=Mus musculus GN=Ywhaz PE=1 SV=1	1433Z	3.83E-02	5.45
Tenascin OS=Mus musculus GN=Tnc PE=1 SV=1	TENA	3.93E-02	1.87
Ig heavy chain V region 6.96 OS=Mus musculus PE=4 SV=1	HVM57	4.02E-02	3.06
Acylamino-acid-releasing enzyme (Fragment) OS=Mus musculus GN=Apeh PE=1 SV=1	A0A0R4J107	4.05E-02	2.31
Early endosome antigen 1 OS=Mus musculus GN=Eea1 PE=1 SV=2	EEA1	4.11E-02	2.76
Proteasome subunit beta type-4 OS=Mus musculus GN=Psmb4 PE=1 SV=1	PSB4	4.22E-02	1.62
Proteasome subunit alpha type-3 OS=Mus musculus GN=Psma3 PE=1 SV=3	PSA3	4.31E-02	1.68
Protein disulfide-isomerase OS=Mus musculus GN=P4hb PE=1 SV=2	PDIA1	4.35E-02	3.73
Cytochrome P450 1A2 OS=Mus musculus GN=Cyp1a2 PE=1 SV=1	CP1A2	4.40E-02	13.40
Annexin A5 OS=Mus musculus GN=Anxa5 PE=1 SV=1	ANXA5	4.40E-02	3.74
Collagen, type VI, alpha 3 OS=Mus musculus GN=Col6a3 PE=1 SV=1	A0A087WS16	4.47E-02	3.85
Protein disulfide-isomerase A3 OS=Mus musculus GN=Pdia3 PE=1 SV=2	PDIA3	4.55E-02	4.50
Immunoglobulin heavy variable V3-4 (Fragment) OS=Mus musculus GN=Ighv3-4 PE=4 SV=1	A0A0A6YXF1	4.57E-02	6.85
MCG23377, isoform CRA_b OS=Mus musculus GN=Gm8797 PE=4 SV=1	A0A0A6YW67	4.61E-02	2.58
Immunoglobulin heavy variable 1-39 (Fragment) OS=Mus musculus GN=Ighv1-39 PE=4 SV=1	A0A075B5V5	4.64E-02	2.27
Tyrosine 3-monooxygenase/tryptophan 5-monooxygenase activation protein, epsilon polypeptide, isoform CRA_c OS=Mus musculus GN=Ywhae PE=1 SV=1	Q5SS40	4.80E-02	4.77
Collagen alpha-1(VI) chain OS=Mus musculus GN=Col6a1 PE=1 SV=1	CO6A1	4.90E-02	6.45
Clathrin heavy chain 1 OS=Mus musculus GN=Cltc PE=1 SV=3	CLH1	4.93E-02	3.10
Proteasome subunit beta type-6 OS=Mus musculus GN=Psmb6 PE=1 SV=3	PSB6	4.94E-02	1.70

Table S5: Full list of blood proteins identified by Progenesis analysis to be differentially abundant between **6- and 12-months** old APP/PS1 mice.

Description	Accession	Anova (p value)	Max fold change
**UPREGULATED (n= 22)**			
Zinc finger protein 280D (Fragment) OS=Mus musculus GN=Zfp280d PE=1 SV=1	V9GXF6	4.36E-04	2.62
CD5 antigen-like OS=Mus musculus GN=Cd5l PE=1 SV=3	CD5L	1.85E-03	2.74
MRP5 (Fragment) OS=Mus musculus GN=Ighv9-4 PE=2 SV=1	Q925S1	2.31E-03	6.21
Translation initiation factor IF-3, mitochondrial (Fragment) OS=Mus musculus GN=Mtif3 PE=1 SV=1	D3YWL2	2.51E-03	43.85
Fibrinogen gamma chain OS=Mus musculus GN=Fgg PE=1 SV=1	FIBG	2.54E-03	2.46
Fibrinogen beta chain OS=Mus musculus GN=Fgb PE=1 SV=1	FIBB	2.99E-03	2.95
Immunoglobulin heavy variable 1-69 OS=Mus musculus GN=Ighv1-69 PE=4 SV=1	A0A075B5X7	4.17E-03	25.59
Fibrinogen alpha chain OS=Mus musculus GN=Fga PE=1 SV=1	FIBA	6.17E-03	2.44
Casein kinase II subunit alpha' (Fragment) OS=Mus musculus GN=Csnk2a2 PE=4 SV=1	A0A1D5RLQ2	7.08E-03	33.06
Immunoglobulin kappa variable 4-80 (Fragment) OS=Mus musculus GN=Igkv4-80 PE=4 SV=2	A0A075B5L7	1.21E-02	3.75
Glycogen phosphorylase, muscle form OS=Mus musculus GN=Pygm PE=1 SV=3	PYGM	1.36E-02	8.03
Major vault protein OS=Mus musculus GN=Mvp PE=1 SV=4	MVP	1.42E-02	2.62
Serine palmitoyltransferase 2 OS=Mus musculus GN=Sptlc2 PE=1 SV=2	SPTC2	1.51E-02	4.25
MCG140437, isoform CRA_d OS=Mus musculus GN=Myh2 PE=1 SV=1	G3UW82	1.82E-02	1.87
Intraflagellar transport protein 172 homolog (Fragment) OS=Mus musculus GN=Ift172 PE=1 SV=1	A0A0J9YU41	2.34E-02	15.28
Anti-norfloxacin MAb light chain variable region (Fragment) OS=Mus musculus PE=2 SV=1	A0A0M3RM39	2.52E-02	42.06
Low density lipoprotein receptor-related protein 1 OS=Mus musculus GN=Lrp1 PE=1 SV=1	A0A0R4J0I9	2.86E-02	1.65
Properdin OS=Mus musculus GN=Cfp PE=2 SV=2	PROP	3.16E-02	1.91
Uncharacterized protein OS=Mus musculus PE=2 SV=1	Q2TAW9	3.76E-02	8.26
Protein-glutamine gamma-glutamyltransferase 2 OS=Mus musculus GN=Tgm2 PE=1 SV=4	TGM2	3.77E-02	4.33
Anti-H5N1 hemagglutinin monoclonal anitbody H5M9 heavy chain (Fragment) OS=Mus musculus PE=1 SV=1	U5LP42	4.44E-02	6.03
Haptoglobin OS=Mus musculus GN=Hp PE=1 SV=1	HPT	4.54E-02	10.76
**DOWNREGULATED (n= 49)**			
T-complex protein 1 subunit gamma OS=Mus musculus GN=Cct3 PE=1 SV=1	TCPG	5.66E-05	Infinity
Kell blood group glycoprotein homolog OS=Mus musculus GN=Kel PE=1 SV=1	KELL	1.62E-03	5.38
Immunoglobulin heavy variable 7-1 (Fragment) OS=Mus musculus GN=Ighv7-1 PE=1 SV=2	A0A075B5S2	2.41E-03	2.01
Proteasome subunit alpha type-6 OS=Mus musculus GN=Psma6 PE=1 SV=1	PSA6	3.37E-03	2.02
Transferrin receptor protein 1 OS=Mus musculus GN=Tfrc PE=1 SV=1	TFR1	5.94E-03	1.88
WD repeat-containing protein 90 OS=Mus musculus GN=Wdr90 PE=1 SV=1	H7BX49	6.15E-03	7.75
Proteasome subunit beta type-3 OS=Mus musculus GN=Psmb3 PE=1 SV=1	PSB3	6.30E-03	2.67
Serglycin OS=Mus musculus GN=Srgn PE=1 SV=1	SRGN	6.31E-03	4.01
Proteasome subunit alpha type OS=Mus musculus GN=Psma7 PE=2 SV=1	Q3TN31	8.31E-03	2.16
Peroxiredoxin-2 OS=Mus musculus GN=Prdx2 PE=1 SV=3	PRDX2	1.01E-02	4.24
Heat shock 70 kDa protein 4 OS=Mus musculus GN=Hspa4 PE=1 SV=1	Q3U2G2	1.06E-02	2.75

26S proteasome non-ATPase regulatory subunit 3 OS=Mus musculus GN=Psmd3 PE=1 SV=3	PSMD3	1.25E-02	4.08
Proteasome subunit beta type-6 OS=Mus musculus GN=Psmb6 PE=1 SV=3	PSB6	1.25E-02	2.17
Bifunctional purine biosynthesis protein PURH OS=Mus musculus GN=Atic PE=1 SV=2	PUR9	1.31E-02	3.79
Uncharacterized protein OS=Mus musculus GN=Psmc5 PE=2 SV=1	A0A1S6GWH1	1.32E-02	23.17
Glyceraldehyde-3-phosphate dehydrogenase OS=Mus musculus GN=Gapdh PE=1 SV=1	A0A0A0MQF6	1.49E-02	2.46
Vimentin OS=Mus musculus GN=Vim PE=1 SV=1	A0A0A6YWC8	1.57E-02	2.37
Clusterin OS=Mus musculus GN=Clu PE=1 SV=1	CLUS	1.59E-02	1.88
Tripeptidyl-peptidase 2 OS=Mus musculus GN=Tpp2 PE=1 SV=3	TPP2	1.74E-02	2.26
Thyrotropin-releasing hormone-degrading ectoenzyme OS=Mus musculus GN=Trhde PE=1 SV=1	TRHDE	1.78E-02	9.31
EG214403 protein OS=Mus musculus GN=Gm4788 PE=2 SV=1	B2RUG5	1.82E-02	2.61
Immunoglobulin heavy variable V1-11 (Fragment) OS=Mus musculus GN=Ighv1-11 PE=4 SV=1	A0A0A6YWI9	1.92E-02	4.34
Proteasome subunit alpha type-3 OS=Mus musculus GN=Psma3 PE=1 SV=3	PSA3	2.16E-02	1.68
Acylamino-acid-releasing enzyme (Fragment) OS=Mus musculus GN=Apeh PE=1 SV=1	A0A0R4J107	2.51E-02	10.22
26S proteasome non-ATPase regulatory subunit 6 (Fragment) OS=Mus musculus GN=Psmd6 PE=1 SV=1	A0A286YDW8	2.75E-02	6.07
Proteasome subunit alpha type OS=Mus musculus GN=Psma5 PE=2 SV=1	Q3TUI9	2.77E-02	2.95
Uncharacterized protein OS=Mus musculus GN=Epb42 PE=2 SV=1	Q3TYT5	2.86E-02	40.43
Carboxypeptidase N catalytic chain OS=Mus musculus GN=Cpn1 PE=1 SV=1	CBPN	3.11E-02	1.71
Integrin alpha 2b OS=Mus musculus GN=Itga2b PE=2 SV=1	B2RPR7	3.13E-02	2.41
26S proteasome non-ATPase regulatory subunit 2 OS=Mus musculus GN=Psmd2 PE=1 SV=1	PSMD2	3.19E-02	2.21
Uncharacterized protein OS=Mus musculus GN=Thbs1 PE=2 SV=1	Q3TR40	3.27E-02	4.69
Ig heavy chain V region 3-6 OS=Mus musculus GN=Ighv3-6 PE=1 SV=1	HVM60	3.31E-02	5.12
Galectin-3-binding protein OS=Mus musculus GN=Lgals3bp PE=1 SV=1	LG3BP	3.32E-02	2.93
26S proteasome regulatory subunit 10B OS=Mus musculus GN=Psmc6 PE=1 SV=1	PRS10	3.44E-02	5.81
Myosin-9 OS=Mus musculus GN=Myh9 PE=1 SV=4	MYH9	3.50E-02	3.36
Glucosidase 2 subunit beta OS=Mus musculus GN=Prkcsh PE=1 SV=1	GLU2B	3.63E-02	6.90
Dihydrolipoyllysine-residue succinyltransferase component of 2-oxoglutarate dehydrogenase complex, mitochondrial OS=Mus musculus GN=Dlst PE=1 SV=1	ODO2	3.83E-02	5.83
Erythrocyte band 7 integral membrane protein OS=Mus musculus GN=Stom PE=1 SV=3	STOM	3.93E-02	6.71
Lumican OS=Mus musculus GN=Lum PE=1 SV=2	LUM	4.00E-02	2.94
Complement component 7 OS=Mus musculus GN=C7 PE=1 SV=2	D3YXF5	4.01E-02	2.35
RIKEN cDNA 1300017J02 gene OS=Mus musculus GN=1300017J02Rik PE=1 SV=1	D3YY36	4.10E-02	2.99
Inter-alpha-trypsin inhibitor heavy chain H2 OS=Mus musculus GN=Itih2 PE=1 SV=1	ITIH2	4.11E-02	1.66
Epidermal growth factor receptor OS=Mus musculus GN=Egfr PE=1 SV=1	EGFR	4.12E-02	3.06
Uroplakin-3b OS=Mus musculus GN=Upk3b PE=1 SV=1	A0A0R4J0S8	4.22E-02	2.01
Metalloproteinase inhibitor 3 OS=Mus musculus GN=Timp3 PE=1 SV=1	TIMP3	4.41E-02	6.47
Large neutral amino acids transporter small subunit 3 OS=Mus musculus GN=Slc43a1 PE=1 SV=1	A2ATS4	4.62E-02	31.86
Aminopeptidase N (Fragment) OS=Mus musculus GN=Anpep PE=1 SV=1	A0A0U1RNS3	4.62E-02	2.47
T-complex protein 1 subunit alpha OS=Mus musculus GN=Tcp1 PE=1 SV=3	TCPA	4.64E-02	3.95
Immunoglobulin heavy variable 1-52 OS=Mus musculus GN=Ighv1-52 PE=4 SV=1	A0A075B5W2	4.79E-02	2.19

Table S6: **Molecular Pathway Enrichment Analysis.** The 37 enriched pathways and the respective differentially abundant proteins involved according to Kyoto Encyclopaedia of Genes & Genomes (KEGG) database using the Enrichr analysis tool. The **p-value** is computed using the Fisher's exact test. The **q-value** is an adjusted p-value using the Benjamini-Hochberg method for correction for multiple hypotheses testing.

Pathway name	Differentially abundant proteins	P-value	q-value
**Proteasome**	PSMB6;PSMA6;PSMB4;PSMC5;PSMA3;PSMB5;PSMC6;PSMA4;PSMD14;PSMC3;PSMD2;PSMD1	5.29E-15	1.63E-12
**Focal adhesion**	ITGB1;LAMA2;ACTN1;ITGB3;ITGA2B;FN1;ILK;LAMC1;THBS1;MYL12A;THBS4;COMP;RAP1B;RAC3;FLNA;FLNB;TLN1;VCL	3.50E-13	5.38E-11
**Phagosome**	ITGB1;TFRC;ITGB3;ITGB2;IGH;CORO1A;THBS1;TUBB4A;THBS4;COMP;EEA1;CANX;CALR;CD36	1.27E-10	1.30E-08
**ECM-receptor interaction**	ITGB1;COMP;LAMA2;ITGB3;ITGA2B;FN1;LAMC1;CD36;THBS1;THBS4	3.89E-09	2.99E-07
**PPAR signaling pathway**	ACSL1;ADIPOQ;PLIN4;ILK;ACSL5;CD36;PLTP;SLC27A5	3.80E-07	2.34E-05
**Regulation of actin cytoskeleton**	ITGB1;ACTN1;ITGB3;ITGA2B;ITGB2;FN1;MSN;RAC3;MYH9;EZR;VCL;MYL12A	6.78E-07	3.48E-05
**PI3K-Akt signaling pathway**	ITGB1;CSF1R;HSP90AA1;LAMA2;ANGPT1;ITGB3;ITGA2B;FN1;LAMC1;IGH;THBS1;THBS4;HSP90B1;COMP;GNB1	9.73E-07	4.28E-05
**Hematopoietic cell lineage**	CSF1R;TFRC;ANPEP;ITGB3;ITGA2B;CD9;CD36;IGH	3.04E-06	1.17E-04
**Leukocyte transendothelial migration**	RAP1B;ITGB1;ACTN1;ITGB2;MSN;EZR;VCL;MYL12A	8.91E-06	3.05E-04
**Proteoglycans in cancer**	ITGB1;ITGB3;FN1;MSN;FLNA;FLNB;EZR;ANK1;THBS1;DCN	1.68E-05	5.19E-04
**Rap1 signaling pathway**	RAP1B;ITGB1;CSF1R;ANGPT1;ITGB3;ITGA2B;ITGB2;RAC3;TLN1;THBS1	2.08E-05	5.83E-04
**Amoebiasis**	LAMA2;ACTN1;ITGB2;FN1;LAMC1;IGH;VCL	2.87E-05	7.38E-04
**Malaria**	COMP;ITGB2;CD36;THBS1;THBS4	8.36E-05	1.98E-03
**Platelet activation**	RAP1B;ITGB1;ITGB3;ITGA2B;TLN1;MYL12A;FERMT3	1.46E-04	3.22E-03
**Dilated cardiomyopathy (DCM)**	ITGB1;LAMA2;ITGB3;ITGA2B;ATP2A2;IGH	1.89E-04	3.89E-03
**Arrhythmogenic right ventricular cardiomyopathy (ARVC)**	ITGB1;LAMA2;ITGB3;ITGA2B;ATP2A2	5.17E-04	9.96E-03
**Epstein-Barr virus infection**	PSMC5;PSMC6;PSMD14;PSMC3;PSMD2;PSMD1;CALR;IGH	5.40E-04	9.79E-03
**Bacterial invasion of epithelial cells**	ITGB1;CLTC;FN1;ILK;VCL	5.87E-04	1.00E-02
**Antigen processing and presentation**	HSP90AA1;HSPA5;HSPA4;CANX;CALR	7.04E-04	1.14E-02
**Complement and coagulation cascades**	ITGB2;MASP2;MASP1;CLU;F5	7.92E-04	1.22E-02
**Human papillomavirus infection**	COMP;ITGB1;LAMA2;ITGB3;ITGA2B;UBR4;FN1;LAMC1;THBS1;THBS4	9.54E-04	1.40E-02
**Tight junction**	ITGB1;HSPA4;ACTN1;MSN;MYH9;EZR;MYL12A	9.84E-04	1.38E-02
**Hypertrophic cardiomyopathy (HCM)**	ITGB1;LAMA2;ITGB3;ITGA2B;ATP2A2	1.10E-03	1.48E-02
**Small cell lung cancer**	ITGB1;LAMA2;ITGA2B;FN1;LAMC1	1.65E-03	2.11E-02
**Fluid shear stress and atherosclerosis**	HSP90AA1;ITGB3;ITGA2B;RAC3;ASS1;HSP90B1	1.78E-03	2.19E-02
**Viral myocarditis**	LAMA2;ITGB2;RAC3;IGH	2.09E-03	2.47E-02
**Ribosome**	RPS9;RPL14;RPL15;RPL18;RPL6;RPL7	2.88E-03	3.28E-02

**Staphylococcus aureus infection**	ITGB2;MASP2;MASP1;IGH	3.50E-03	3.85E-02
**Glycolysis / Gluconeogenesis**	LDHA;DLAT;BPGM;GAPDH	3.50E-03	3.72E-02
**Adipocytokine signaling pathway**	ACSL1;ADIPOQ;ACSL5;CD36	3.69E-03	3.79E-02
**Protein processing in endoplasmic reticulum**	HSP90AA1;HSPA5;PRKCSH;CANX;CALR;HSP90B1	4.17E-03	4.14E-02
**Starch and sucrose metabolism**	MGAM;UGP2;PYGM	4.31E-03	4.15E-02
